# Bimodal Expression Patterns, and Not Viral Burst Sizes, Predict the Effects of Vpr on HIV-1 Proviral Populations in Jurkat Cells

**DOI:** 10.1128/mbio.03748-21

**Published:** 2022-04-06

**Authors:** Edmond Atindaana, Abena Kissi-Twum, Sarah Emery, Cleo Burnett, Jake Pitcher, Myra Visser, Jeffrey M. Kidd, Alice Telesnitsky

**Affiliations:** a Department of Microbiology and Immunology, University of Michigan Medical School, Ann Arbor, Michigan, USA; b West African Centre for Cell Biology of Infectious Pathogens (WACCBIP), University of Ghana, Legon, Greater Accra Region, Ghana; c Department of Biochemistry, Cell and Molecular Biology, University of Ghana, Legon, Greater Accra Region, Ghana; d Human Genetics, University of Michigan Medical School, Ann Arbor, Michigan, USA; Rutgers-Robert Wood Johnson Medical School

**Keywords:** HIV-1 expression properties, HIV-1 persistence, HIV-1 population dynamics, integration sites, latency reactivation, latency-reversing agents, LRAs

## Abstract

Integration site landscapes, clonal dynamics, and latency reversal with or without *vpr* were compared in HIV-1-infected Jurkat cell populations, and the properties of individual clones were defined. Clones differed in fractions of long terminal repeat (LTR)-active daughter cells, with some clones containing few to no LTR-active cells, while almost all cells were LTR active for others. Clones varied over 4 orders of magnitude in virus release per active cell. Proviruses in largely LTR-active clones were closer to preexisting enhancers and promoters than low-LTR-active clones. Unsurprisingly, major *vpr*^+^ clones contained fewer LTR-active cells than *vpr*^−^ clones, and predominant *vpr*^+^ proviruses were farther from enhancers and promoters than those in *vpr*^−^ pools. Distances to these marks among intact proviruses previously reported for antiretroviral therapy (ART)-suppressed patients revealed that patient integration sites were more similar to those in the *vpr*^+^ pool than to *vpr*^−^ integrants. Complementing *vpr*-defective proviruses with *vpr* led to the rapid loss of highly LTR-active clones, indicating that the effect of Vpr on proviral populations occurred after integration. However, major clones in the complemented pool and its *vpr*^−^ parent population did not differ in burst sizes. When the latency reactivation agents prostratin and JQ1 were applied separately or in combination, *vpr*^+^ and *vpr*^−^ population-wide trends were similar, with dual-treatment enhancement being due in part to reactivated clones that did not respond to either drug applied separately. However, the expression signatures of individual clones differed between populations. These observations highlight how Vpr, exerting selective pressure on proviral epigenetic variation, can shape integration site landscapes, proviral expression patterns, and reactivation properties.

## INTRODUCTION

HIV-1 establishes stable reservoirs in patients treated with antiretroviral therapy (ART), which consist of cells containing replication-competent proviruses that are not cleared by the immune system and that can rekindle spreading infection (1). It is generally assumed that the latent reservoir consists of proviruses that are transcriptionally silent, but what causes this is unclear. Reservoir establishment and maintenance are multifaceted and may involve the intracellular depletion of transcription factors, viral regulatory protein deficiencies, provirus integration position effects, and epigenetic variation (2–12). The oligoclonal nature of ART-suppressed patients’ proviruses suggests that the reservoir’s long-lived nature is at least partially due to infected cells’ proliferation, which may be either homeostatic or driven by T cell receptor engagement (13–15).

Fundamental aspects of latency in patients, such as the size of the latent reservoir, remain poorly defined. Method improvement for reservoir quantification is ongoing, as recent studies have demonstrated that quantitative viral outgrowth assays (QVOAs) underestimate the latent reservoir, while PCR-based quantification can overestimate it due to the predominance of defective proviruses (16–18). Experimental approaches for reactivation must sometimes be applied multiple times to achieve reactivation *ex vivo* (19). Moreover, reported genetic dissimilarities between *ex vivo* outgrowth virus and reemergent viremia suggest that existing reactivation approaches may not accurately detect rebound-competent virus (20, 21). Large interclonal differences in virion release per T cell and variability in virion release upon latency disruption may further complicate reservoir size determination (22, 23).

The rarity of latently infected cells in virally suppressed individuals makes the study of *in vitro* latency models necessary. Some tissue culture models for HIV-1 latency and reactivation, such as the widely used J-Lat clones, assess long terminal repeat (LTR) promoter activity by reporter gene expression but lack genetic elements believed to be dispensable for HIV-1 latency (24–26). One such element is the *vpr* gene, whose product plays roles in viral infectivity *in vivo* (27–30) but also causes cell cycle arrest and can induce widespread changes in host gene expression (31–33). Many latency models use *vpr*-defective proviruses, which, when cultured over time to allow proviral silencing, can be used for reactivation studies (34–37). However, in such systems, the extent to which proviral quiescence represents the silencing and outgrowth of a subset of integrant clones versus global proviral silencing is unknown.

Cell-based latency models have been used to pilot candidate cure strategies, including “shock-and-kill” and “block-and-lock” approaches. In contrast to ART, which prevents viral spread, the shock-and-kill method involves inducing virus expression with the intention that this will lead to either cytopathic death of reactivated cells or host immune recognition and infected cell clearance (38). Candidate latency reactivation approaches that perturb cellular pathways or complement intracellular deficiencies in experimental models include the use of prostratin, which stimulates T cells without inducing cellular proliferation and increases the level of NF-κB (39, 40). Other latency-reversing agents (LRAs) include those that act to increase the level of pTEFb, including the BET bromodomain inhibitor JQ1 (41), as well as treatments that modify the chromatin environment, such as histone deacetylase inhibitors like suberoyl anilide hydroxamic acid (SAHA) and entinostat (42–46). In contrast, the block-and-lock approach seeks to permanently silence proviruses to prevent their reactivation (47).

Molecules that are effective at reversing latency in various tissue culture models have been identified, but evidence thus far suggests either that these are too toxic to be therapeutically useful or that they fail to reduce the size of the latent reservoir in patients (46, 48–51). This discordance may be due in part to differences among cell-based latency models, as some use infectious virus while others use viral reporters, and some use clonal integrants while others use heterogeneous proviral populations (25). Thus, the inconsistent results in LRA reactivation studies using these models may reflect that each model captures at best a subset of the expression properties that contribute to the latent reservoir (52), and whereas the effects on heterogeneous populations of integrants have been examined extensively, the contributions of individual clones’ behavior to aggregate population responses largely have not been explored.

In the current study, the HIV-1 expression properties of hundreds of individual integrant clones were compared within polyclonal populations of barcoded proviruses. The influences of integration sites and the presence or absence of *vpr* on the populations’ clonal structures and their reactivation dynamics were investigated. Together, these findings suggest that Vpr’s cytotoxic activity disproportionately affects a distinct subset of infected cells and plays critical roles in both shaping transcriptionally inactive proviral populations and defining their reactivation potential.

## RESULTS

### *vpr*^−^ and *vpr*^+^ proviral pools differed in their numbers of LTR-active cells.

**FIG 1:**
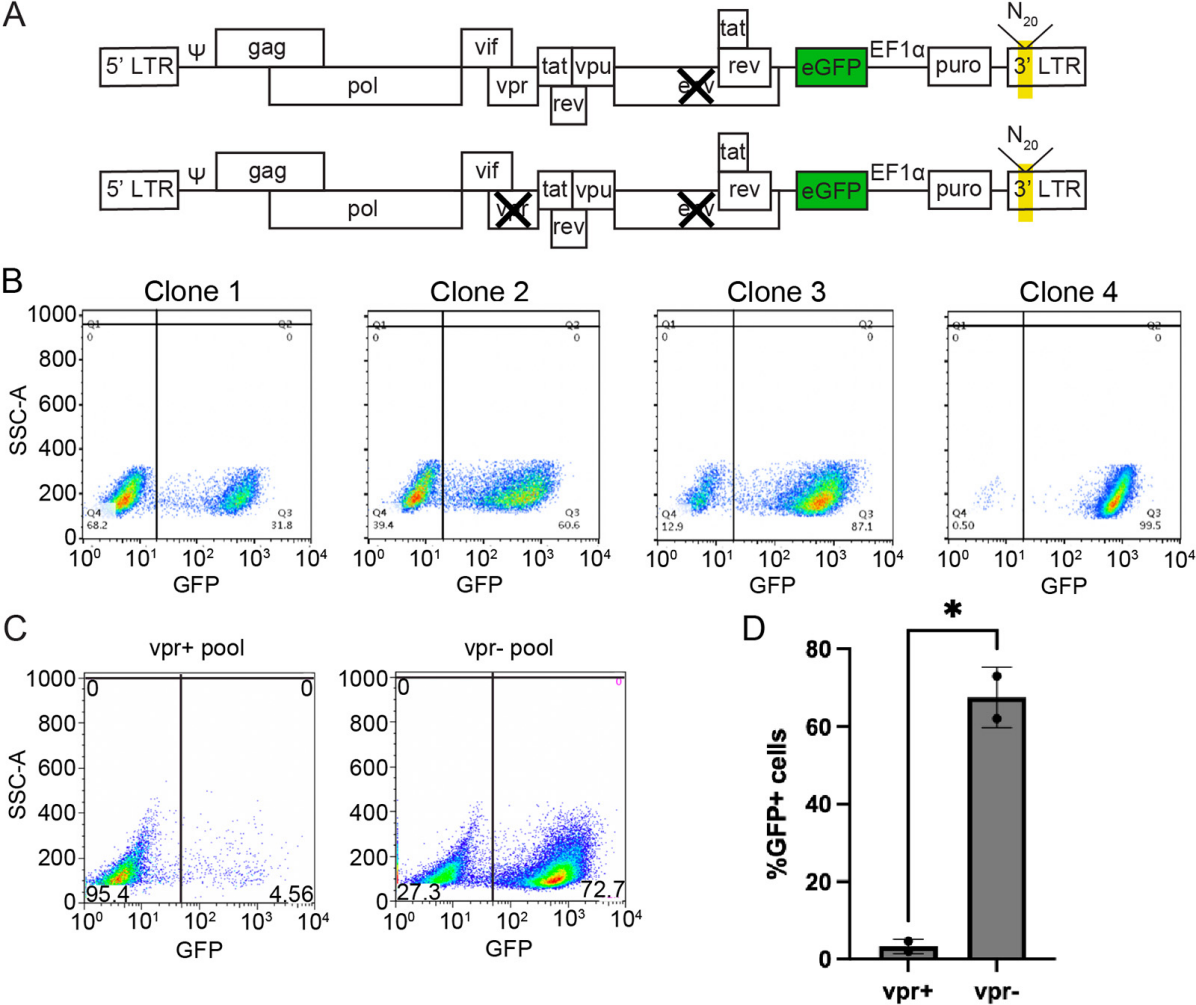
Generation of zip-coded HIV-1 proviral pools. Polyclonal cell pools containing *vpr*^+^ and *vpr*^−^ proviruses were established in Jurkat T cells and compared. (A) Schematic of vectors used to produce zip-coded virus (not to scale). The yellow shading in the 3′ LTR indicates the position of the 20-bp randomized barcode in U3. eGFP, enhanced green fluorescent protein. (B) *vpr*^−^ clone expression properties. Three clones were isolated by limiting dilution of *vpr*^−^ pool cells, expanded for 14 days, and subjected to flow cytometry. The flow plots show that clonal pools 1 to 3 have different percentages of GFP^+^ cells. The *x* axis indicates the GFP signal detected in the FITC channel; numbers in each gate indicate the percentages of cells gated GFP^−^ or GFP^+^. SSC, side scatter. (C) Representative flow cytometry plots of polyclonal Jurkat T cells generated by infection with barcoded *vpr*^+^ (left) or *vpr*^−^ (right) viruses that were puromycin selected for 4 days and expanded for an additional 10 days. (D) Bar graph comparing the percentages of GFP^+^ puromycin-resistant cells in polyclonal *vpr*^+^ and *vpr*^−^ populations. Data represent results for two *vpr*^+^ pools and two *vpr*^−^ pools (* indicates a *P* value of <0.05 by a paired *t* test).

To study the effects of Vpr on the responsiveness of individual proviruses in the absence of virus spread, Jurkat cells were infected with distinguishable *vpr*-positive (*vpr*^+^) or *vpr*-negative (*vpr*^−^) versions of the NL4-3 derivative shown in Fig. 1A (53). The use of the EF1α promoter to drive the constitutive expression of the puromycin resistance gene allowed the selection of infected cells independent of LTR expression. Each genomic RNA in an infecting virus contained a unique 20b randomized “barcode” inserted into U3, which was duplicated in both LTRs after reverse transcription and enabled the tracking of individual proviruses. We refer to the barcodes as “zip codes” because in the context of proviruses, they report the genetic neighborhoods of each integrant. Infected cell populations were passaged without cell cloning, thus generating polyclonal integrant populations within which transcriptionally active cells were identified using LTR-driven green fluorescent protein (GFP) expression or by progeny genomic RNA released in Env^−^ virions.

It has long been recognized that at least some HIV-1 integrants establish clones in which a fraction of the total daughter cells possess active LTRs while proviruses are silenced in other sibling cells (54). Previous work using zip-coded *vpr*^−^ derivatives of the vector used here has shown that for each integrant, the clone gives rise to a mixture of cellular progeny that includes some GFP^+^ cells and some GFP^−^ cells and that over time sibling cells switch between LTR-active (GFP^+^) and -inactive (GFP^−^) expression phenotypes while maintaining LTR-active and -inactive cells in stable equilibrium proportions (53). To confirm that individual integrant clones in the current study also contained clone-specific mixtures of GFP^+^ and GFP^−^ cells, single cells were isolated by limiting dilution from the *vpr*^−^ pool and expanded, and cells from each clone were then subjected to flow cytometry (Fig. 1B). Consistent with previously reported differences in bimodal expression patterns among proviral clones (53), the results showed that each clonal pool was comprised of both GFP^+^ and GFP^−^ cells, with LTR-active proportions being discernible by GFP^+^ percentages (%GFP^+^) that differed among the clones: mostly GFP^+^ cells for some clones (high-%GFP^+^ clones) and distinctly different GFP^+^ percentages for others (Fig. 1B). To confirm that these cells were clonal and contained single proviruses and, thus, that the observed mixed cell phenotypes did not reflect the presence of more than one clone, PCR amplicons from these clones were Sanger sequenced without subcloning and determined to display unique sequences, indicating that at least a large majority of each clone’s cells contained a unique zip code (see Materials and Methods).

In the current study, four independent polyclonal integrant pools were established by infecting roughly 5 × 10^6^ Jurkat T cells at a low multiplicity of infection (<0.0005) to ensure that puromycin-resistant infected cells contained only one provirus per cell. Two of the pools contained *vpr*^+^ proviruses, and two had integrants lacking *vpr*. Flow cytometry analysis after 14 days of expansion showed significantly fewer GFP^+^ cells in the two *vpr*^+^ infected cell pools than in the two *vpr*^−^ provirus pools (<5% versus 73% GFP^+^, respectively; *P* = 0.042 by a paired *t* test) (Fig. 1C and D). To address the possibility that uninfected cell outgrowth might have contributed to GFP^−^ cell populations, an uninfected Jurkat cell control was cultured in parallel and subjected to the same schedule of puromycin treatment and selection-free medium exposure as that for the infected cells. After a total of 14 days, these uninfected control cultures were analyzed by flow cytometry to ensure that no surviving uninfected cells were detectable that might confound observations. In an additional test of the possible presence of uninfected cells, dual aliquots of each pool were analyzed about 30 days after pool establishment. One aliquot was cultured with puromycin, and the other was left untreated. When analyzed by propidium iodide (PI) staining for cell death, no difference was observed between treated and untreated pool controls.

At 14 days postinfection, *vpr*^−^ and *vpr*^+^ cell pools were each sorted into GFP^+^ and GFP^−^ subpools, and cellular DNA was harvested from an aliquot of each subpool immediately after sorting. The integrants’ zip codes were amplified from the cellular DNA samples in at least two separate PCRs per cellular DNA sample, and the subpools’ zip code contents were catalogued by high-throughput sequencing. Correlation analysis for the fractional abundances of specific zip codes in replicate reactions showed reproducible zip code detection (see Fig. S1 in the supplemental material).

10.1128/mbio.03748-21.1 FIG S1 Reproducible amplification and high-throughput sequencing of zip codes. Two independent PCRs were set up to generate amplicon zip codes from gDNA samples and high-throughput sequenced. The relative abundances of zip codes were quantified, and the log_10_ (relative abundance) values of PCR amplicon 1 (replicate 1) and PCR amplicon 2 (replicate 2) were plotted on the *x* and *y* axes, respectively. The color bar indicates the log_10_ relative abundances of zip codes in replicate 2. The darker the blue scale, the more abundant the zip code. Download FIG S1, TIF file, 0.4 MB. Copyright © 2022 Atindaana et al. 2022 Atindaana et al. https://creativecommons.org/licenses/by/4.0/ This content is distributed under the terms of the Creative Commons Attribution 4.0 International license.

After analyzing roughly 3 million sequencing reads per pool, zip codes were ordered by read abundance. Determining how many unique barcodes were present in each GFP^+^ pool revealed that similar numbers of zip codes (approximately 2,000), each indicative of a single integration event, were detected in the GFP^+^ sorted cells from both the *vpr*^+^ and *vpr*^−^ pools, even though the GFP^+^ cell fraction of the *vpr*^+^ pool was very small. Because all four pools had been infected at the same multiplicity of infection, this finding suggested that the numbers of GFP^+^ integrants initially established did not differ markedly between pools, which is consistent with previous work in dendritic cells that indicates that the extent of proviral integration does not differ depending on the presence or absence of Vpr (55).

**FIG 2:**
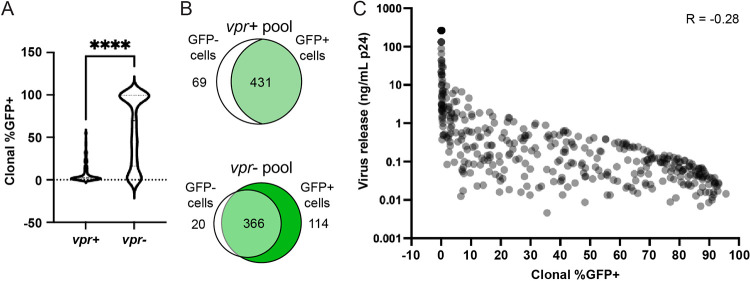
Expression properties of individual clones within the *vpr*^+^ and *vpr*^−^ pools. (A) Comparison of the numbers of high- and low-LTR-active clones in *vpr*^+^ and *vpr*^−^ pools. The fractions of total cells in each clone that sorted GFP^+^ (LTR active) were calculated for the 500 most abundant clones in each pool by quantifying the fraction of each zip code (integrated barcode) in the GFP^+^ sorted cells’ genomic DNA to its quantity in the unsorted pool by high-throughput sequencing and normalizing for the percentage of the unsorted pool that was GFP^+^ (see Materials and Methods). Violin plots compare the %GFP^+^ values (indicative of the clones’ fraction of LTR-active daughter cells) for the top 500 most abundant zip codes in each pool (**** indicates a *P* value of <0.0001 by a Mann-Whitney U two-tailed test). (B) Venn diagrams of the numbers of independent zip codes in GFP^+^ and GFP^−^ sorted subpools for *vpr*^+^ and *vpr*^−^ pools. The number in the white region indicates the number of different zip codes observed only in GFP^−^ sorted cells, that in the light-green region of the intersection represents the number of zip codes that were present in both GFP^+^ and GFP^−^ sorted subpools, and the number in the dark-green region represents zip codes present in only the green sorted cells. The 500 reported zip codes in each pool represent those present in the top 78% and 88% of reads for *vpr*^+^ and *vpr*^−^ pools, respectively, when clones were ordered by read abundance. (C) Scatterplot displaying each clone’s %GFP^+^ value on the *x* axis and virus release per GFP^+^ cell on the *y* axis (Pearson’s *R* = −0.28).

Combining population-wide percentages of GFP^+^ cells in the unsorted cells, as determined by flow cytometry, with zip code read counts within sorted subpool libraries allowed calculating the percentage of LTR-active cells (%GFP^+^ values) within each cell clone (Fig. 2A). The clones’ %GFP^+^ values were calculated using zip code abundances in fluorescence-activated cell sorter (FACS)-sorted GFP^+^ and GFP^−^ cells’ DNA and normalizing the values to population-wide GFP positivity levels at sort time, as described in Materials and Methods. Notably, for the top 500 most abundant clones in the unfractionated *vpr*^+^ and *vpr*^−^ pools, the median %GFP^+^ value was significantly lower in the *vpr*^+^ pool (*P* = 0.0001 by a Mann-Whitney U two-tailed test), indicating that most cells in the *vpr*^+^ pool were members of low-LTR-active clones (Fig. 2A). When GFP^+^ sorted cells from *vpr*^+^ and *vpr*^−^ pools were cultured further, no viable *vpr*^+^ GFP^+^ cells were detected after 3 days, and thus, although integrated zip codes in the freshly sorted cells were determined, the expression properties and clonal structures of *vpr*^+^ GFP^+^ cells could not be analyzed further. These results indicated that integration events that resulted in GFP expression were equally likely in *vpr*^+^ and *vpr*^−^ proviral pools when the cells were examined early after infection. However, the depletion of the *vpr*^+^ GFP^+^ cell subpopulations suggested that whenever cells with *vpr*^+^ proviruses switched phenotypes from LTR inactive to LTR active, the resulting GFP^+^ cells died upon further cell culturing.

### Vpr shaped the clonal structure of infected pools.

The observation of fewer GFP^+^ cells in the *vpr*^+^ pool than in cells with *vpr*^−^ proviruses was not surprising due to Vpr’s well-known cytotoxic effects. Along with *env*, *tat*, *nef*, and *vpu*, *vpr* is one of the HIV-1 genes reported to be cytotoxic in at least some contexts (56–58). However, despite Vpr’s cytotoxicity, a small fraction of *vpr*^+^ GFP^+^ cells was observed among unsorted *vpr*^+^ cells, even though sorted *vpr*^+^ GFP^+^ cells did not survive 3 days of culture.

One plausible reason why unsorted *vpr*^+^ populations contained rare GFP^+^ cells was that these cells arose via recent phenotypic switches from clones that were largely inactive. To test this possibility, zip codes in GFP^+^ and GFP^−^ sorted subpools were compared for both *vpr*^+^ and *vpr*^−^ integrants (Fig. 2B). Ordering zip codes by their abundance in the unsorted pools and analyzing those that comprised the top 500 revealed that among *vpr*^−^ cells, about 73.2% of all the zip codes were found in both subpools, while 4% were observed only in GFP^−^ cells and 22.8% were observed only in the GFP^+^ subpool. In contrast, 86.2% of the *vpr*^+^ cells’ zip codes were found in both subpools, with 13.8% being observed only in the GFP^−^ subpool and none of the zip codes being found exclusively in the GFP^+^ subpool. This suggested that the small fraction of GFP^+^ cells in the *vpr*^+^ pool (Fig. 1C and D) resulted from the recent acquisition of LTR expression by cells from the larger GFP^−^ cell pool. If the *vpr*^−^ pool is assumed to be relatively representative of a population that results when all clones are equally viable, this suggests that when initially integrated, proviruses whose daughter cells were largely or always GFP^+^ were the dominant class of clones.

### Clones differed in LTR-active cell burst sizes.

Because the proviral vectors used in this study contained most HIV-1 genes and expressed packageable RNAs as well as a GFP reporter, expression properties could be measured by both GFP expression and virion release. To determine the amount of virus released per LTR-active cell in each clone, total virus release from the *vpr*^−^ GFP^+^-fractionated cell pool was quantified by p24 release; the limited viability of the *vpr*^+^ GFP^+^ cell fraction prevented this analysis for the *vpr*^+^ pool. Relative zip code amounts in virion RNA and in genomic DNA (gDNA) extracted from the GFP^+^ cells were then determined and used to calculate the amount of virus released per LTR-active cell for each clone. These results are indicated in Fig. 2C, with each clone’s release per LTR-active cell presented on the *y* axis and clones ordered by their member cells’ %GFP^+^ values on the *x* axis. All clones in Fig. 2C are represented in light gray; regions that appear to display darker shading indicate the presence of multiple clones at the same coordinates. We and others have previously reported significant differences among HIV-1-infected cell clones in the amount of virus released per active cell (23, 53), and the results here indicated that burst sizes for the integrant clones in the current study ranged over 4 orders of magnitude.

Notably, burst sizes were slightly negatively correlated with %GFP^+^ values (Pearson’s *R* = −0.28) (Fig. 2C), indicating that clones that displayed higher %GFP^+^ values tended to produce fewer virions per LTR-active cell than low-%GFP^+^ clones. Because the intactness of these proviruses was not addressed directly, it is possible that some of the observed variation in virus release reflected provirus defects. However, because this work quantified encapsidated viral RNAs, even low-virus-expressing proviruses must have remained largely intact, as proviruses with large internal deletions would have lost the ability to assemble viral particles. Another possible contributor to these differences may be that large burst sizes conferred some selection against high-LTR-active clones during the weeks of infected cell passage that preceded these measurements. Although Vpr, which was not present in these cells, is the HIV-1 protein best recognized as cytotoxic, the expression of the retained HIV-1 proteins or other elements may attenuate high-%GFP^+^ clones in more subtle ways.

### High-LTR-active clones’ proviruses were in closer proximity to genome marks associated with active gene expression.

**FIG 3:**
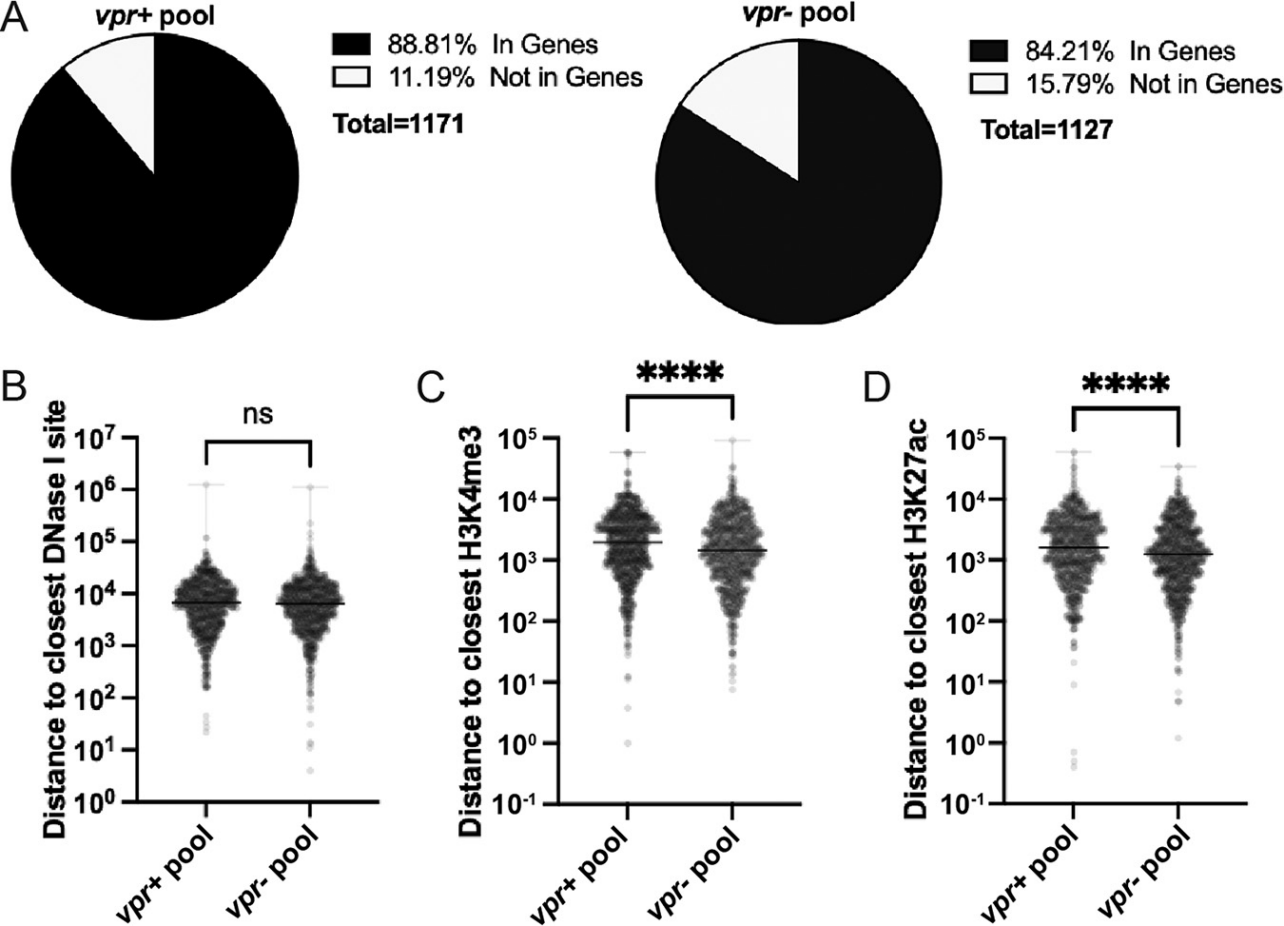
Comparison of integration sites in *vpr*^+^ and *vpr*^−^ pools. Integration sites for both *vpr*^+^ and *vpr*^−^ pool cells harvested at 10 days postinfection were determined, and distances to the nearest indicated genome features were mapped and compared. (A) Pie charts showing the percentage of integration sites found within genes versus those not found in genes for *vpr*^+^ and *vpr*^−^ pools. (B to D) Box plots showing pairwise comparisons between *vpr*^+^ and *vpr*^−^ integration sites’ distances to the closest DNase I hypersensitivity site (means = 10,687 and 10,236, respectively) (B), H3K4me3 (means = 32,989 and 27,047, respectively) (C), and H3K27ac (means = 28,485 and 22,228, respectively) (D). A Mann-Whitney U two-tailed test was conducted for pairwise comparisons for panels B to D (ns [nonsignificant] and **** indicate *P* values of >0.05 and <0.0001, respectively) (*n* = 1,171 and 1,127 for *vpr*^+^ and *vpr*^−^, respectively).

The finding that LTR-active *vpr*^+^ cells were rapidly lost suggested that proviruses that had integrated into more active regions of the host genome might be selected against within polyclonal *vpr*^+^ populations. To examine this notion, we compared the proximities of *vpr*^+^ and *vpr*^−^ integration sites to genomic features associated with active gene expression. First, zip code integration sites were determined using cellular DNA harvested 10 days after the establishment of each pool and mapped to 1,171 and 1,121 unique sites in the human genome for *vpr*^+^ and *vpr*^−^ pool members, respectively (see Materials and Methods). Next, the proportions of zip codes located in genes versus those not in genes (as defined by ENCODE for Jurkat cells [59]) were compared (see Fig. 3A and Fig. S2 for the first and duplicate pools, respectively). Consistent with previous reports (60–62), the results indicated that similar majorities of integrants were established within genes regardless of whether or not *vpr* was present. Next, *vpr*^+^ and *vpr*^−^ integrants were compared for their proximities to specific genome marks associated with active gene expression that have been reported to preexist in Jurkat cells (63). No differences were found in distances to the closest DNase I sensitivity sites, which are associated with open chromatin (*P* = 0.1854 by a Mann-Whitney U test) (Fig. 3B). However, the proximities to H3K27ac (associated with enhancers) (*P* < 0.0001 by a Mann-Whitney U two-tailed test) and H3K4me3 (associated with active promoters) (*P* < 0.0001 by a Mann-Whitney U two-tailed test) marks differed significantly, with proviruses from *vpr*^+^ pools being somewhat farther from these marks (Fig. 3C and D).

10.1128/mbio.03748-21.2 FIG S2 Comparison of integration sites and %GFP^+^ values between duplicate *vpr*^−^ and *vpr*^+^ pools. (A) Integration sites were sequenced and mapped to genes in Jurkat T cells. A total of 92% of integration sites were found in genes and 8% were not found in genes for *vpr*^+^ pool 2 (*n* = 166), compared to 90% within genes and 10% not within genes for *vpr*^−^ pool 2 (*n* = 67). Download FIG S2, TIF file, 0.8 MB. Copyright © 2022 Atindaana et al. 2022 Atindaana et al. https://creativecommons.org/licenses/by/4.0/ This content is distributed under the terms of the Creative Commons Attribution 4.0 International license.

**FIG 4:**
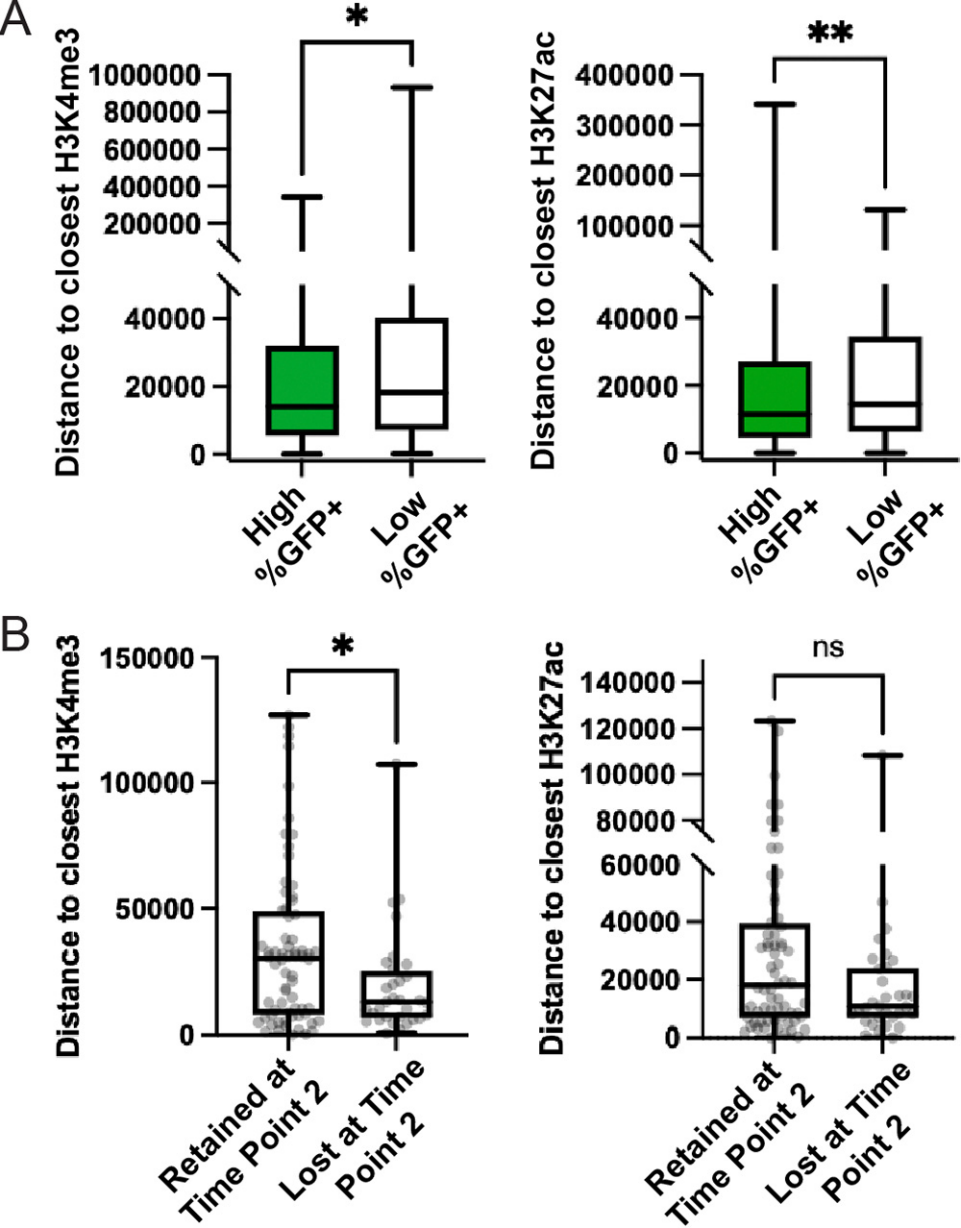
Integration site proximities to chromatin marks and their changes over time in *vpr*^+^ pools. (A) Distances to the indicated marks for high- and low-%GFP^+^ clones. The %GFP^+^ values for each zip code from the *vpr*^−^ pool were determined as described in Materials and Methods. Zip codes with %GFP^+^ values of ≥60% were binned as “high-LTR-active clones” (595 zip codes), and zip codes with %GFP^+^ values of <30% were binned as “low-LTR-active clones” (222 zip codes). Pairwise comparisons of the distances to the closest H3K4me3 (left) and H3K27ac (right) marks are shown. (B) Changes in predominant integration site features for the *vpr*^+^ pools over time. Among the top 100 most abundant zip codes in the unsorted *vpr*^+^ pool at time point 1 (10 days postinfection), 68 were retained among the top 100 at time point 2 (24 days postinfection), while the remaining 32 time point 1 top 100 clones were lost at time point 2 and supplanted by other, formerly less-abundant zip codes. Box plots compare the closest H3K4me3 or H3K27ac mark for the 68 time point 1 zip codes that were retained in the top 100 at time point 2 to the 32 time point 1 zip codes that had been lost at time point 2 (ns indicates a *P* value of >0.05 by a Mann-Whitney U two-tailed test).

To correlate the clones’ LTR-active proportions with their proximities to active chromatin marks, distances from these marks were compared for high- and low-LTR-active clones. Few clones displayed intermediate %GFP^+^ levels (Fig. 2A), and all major clones in the *vpr*^+^ pool had clonal %GFP^+^ proportions of 30% or lower. Therefore, only integrants from the *vpr*^−^ pool were examined, and low-LTR-active clones (%GFP^+^ values of <30%) were compared to high-LTR-active clones (%GFP^+^ values of ≥60%) (Fig. 4A). This analysis showed that the distances to both H3K27ac and H3K4me3 marks were shorter among high-LTR-active clones than among low-LTR-active clones, albeit not dramatically, suggesting that the difference in integration site proximities observed between *vpr*^+^ and *vpr*^−^ proviruses may be due to the survival of high-LTR-active clones in the absence of Vpr.

Because the *vpr*^+^ pool was devoid of high-LTR-active clones, its high- and low-LTR-active integrants could not readily be compared. However, with the reasoning that the abundances of residual high-LTR-active clones would gradually diminish over time, the dynamics of the v*pr*^+^ pool were examined by comparing samples harvested 2 weeks apart. Examination of the 100 most abundant zip codes in the unsorted *vpr*^+^ cell pool showed that only 68 of the top 100 zip codes present at day 10 postinfection (time point 1) were observed among the 100 most abundant zip codes on day 24. When integration site proximities to H3K4me3 and H3K27ac marks were compared, the 32 zip codes that were absent from the top 100 on day 24 (time point 2) (Fig. 4B) were significantly closer to H3K4me3 (*P* = 0.0193 by a Mann-Whitney U two-tailed test) and tended to be closer to H3K27ac marks (although not with statistical significance [*P* = 0.1581 by a Mann-Whitney U two-tailed test]) than the 68 time point 1 clones that remained within the top 100 most abundant clones at time point 2.

### The spectra of LRA responses were similar for vpr^+^ and vpr^−^ populations, but clonal behaviors differed.

**FIG 5:**
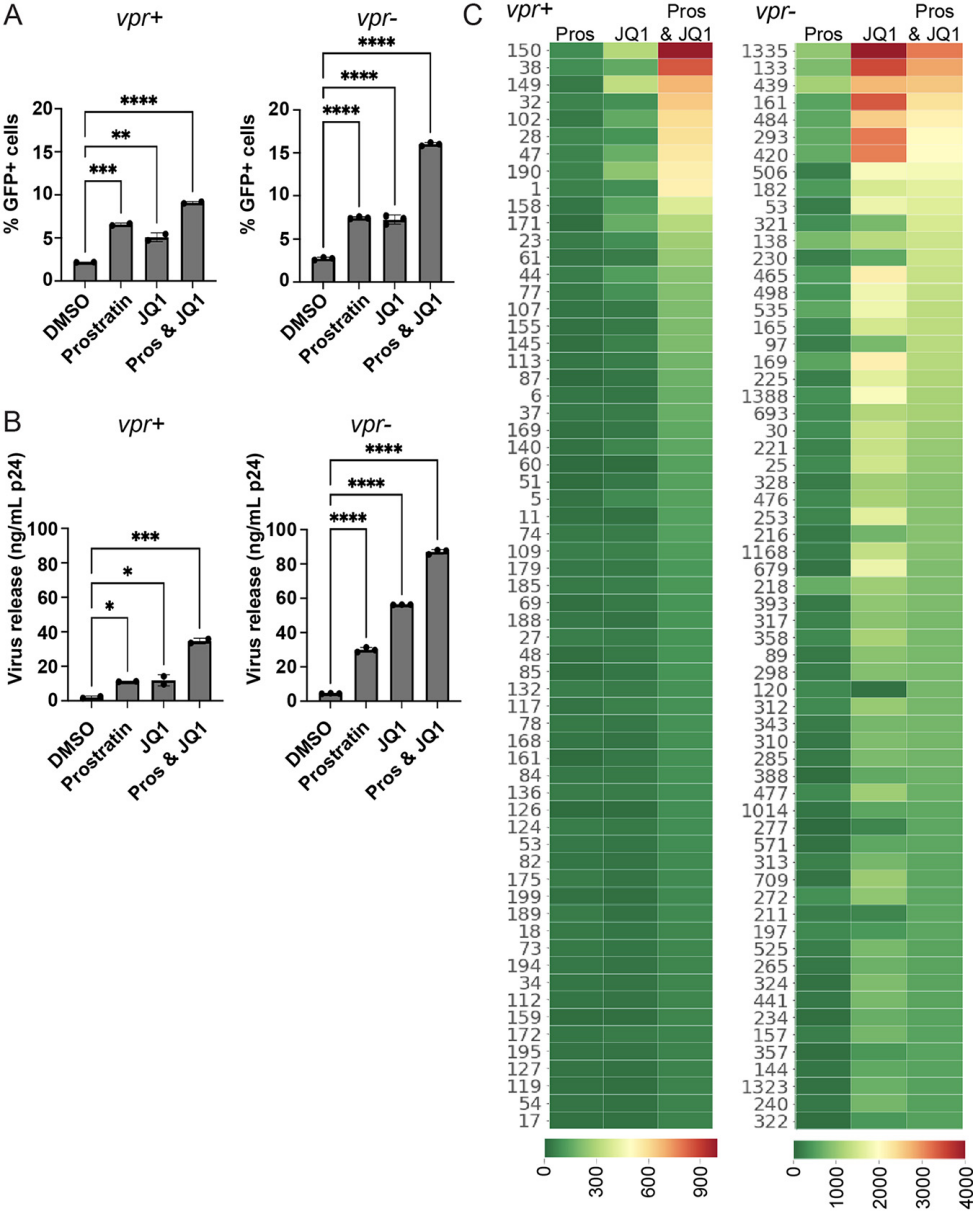
Effects of LRAs on zip-coded pools and clones. *vpr*^+^ and *vpr*^−^ GFP^−^ cell fractions were exposed to 0.1% DMSO, 2 μM JQ1, 2 μM prostratin, or a combination of prostratin and JQ1. Reactivation to GFP^+^ was measured at 24 h postinfection by flow cytometry, and virus release was quantified using a reverse transcriptase assay and normalized to define p24 levels. (A and B) Bar graphs showing the frequency of GFP^+^ cells after LRA treatment (left panel for each pool) (A) and the amount of virus released into the culture supernatant (right panel for each pool) (B) for the indicated polyclonal pools. The error bars show the means and standard deviations from two experimental treatment repetitions. (C) Heat maps of the clonal (zip code) virus release per treated GFP^−^ cell (left, *vpr*^+^ pool; right, *vpr*^−^ pool). Numbers at the left of each panel are clone identifiers generated by ordering proviral zip codes in decreasing relative abundances, as determined for the unsorted pools. Every row represents a unique cell clone’s response. The clones were ordered from top to bottom by diminishing virus release per treated cell upon dual-LRA treatment. The color bar indicates the extent of release per treated cell based on p24 values in arbitrary units. Note that the same unit values were used for both *vpr*^+^ and *vpr*^−^ pools but that the color scales, as presented at the bottom of the panels, differ between pools.

To address possible functional consequences of *vpr*, the reactivation properties of LTR-inactive *vpr*^−^ and *vpr*^+^ subpools were compared. The LRAs prostratin, a protein kinase C agonist, and JQ1, a bromodomain inhibitor, were applied separately or in combination to the GFP^−^ subpopulations of each pool for 24 h. Reactivation for each treated GFP^−^ population was monitored by both determining the changes in the frequency of GFP^+^ cells, as measured by flow cytometry, and quantifying virus release (Fig. 5A and B; Fig. S3A and B). The results indicated that compared to single prostratin and JQ1 treatments, dual treatment resulted in additive levels of reactivation in both *vpr*^+^ and *vpr*^−^ populations by both criteria (Fig. 5A shows reactivation monitored by GFP^+^ cells, and Fig. 5B shows virus release [the left panel indicates reactivation for *vpr*^+^, and the right panel shows reactivation for *vpr*^−^]). In dual-LRA treatments for both pools, there was an ∼4-fold increase in GFP^+^ cells, while virus release increased by ∼30-fold relative to the dimethyl sulfoxide (DMSO) control. The most significant difference between the *vpr*^−^ and *vpr*^+^ pools was that the absolute amount of virus release upon treatment was 3-fold higher in the *vpr*^−^ pools, and the responsiveness to JQ1 was lower in the *vpr*^+^ pools. These differences between pools in their extents of reactivation were not due to differences in cell viability (Fig. S4C). The observation that reactivation was enhanced by dual prostratin and JQ1 treatment is consistent with previous works by Boehm et al. and Darcis et al. using the same drugs in cell culture models of latency and *ex vivo*-treated cells from HIV-1 patients, respectively (24, 41).

10.1128/mbio.03748-21.3 FIG S3 **Cell viability and reactivation of additional *vpr*^−^ and *vpr*^+^ pools.** *vpr*^+^ and *vpr*^−^ GFP^−^ cells were exposed to 0.1% DMSO, 2μM JQ1, 2μM prostratin, and a combination of prostratin and JQ1. Reactivation was measured at 24 hours post-infection by flow cytometry, and virus release was quantified by reverse transcriptase assay. (A) Bar graphs showing the frequency of GFP^+^ cells post LRA treatment (left panel for *vpr*^+^ and right panel for *vpr*^−^). (B) The amount of virus released into culture supernatant (left panel for *vpr*^+^ and right panel for *vpr*^−^) for the indicated polyclonal pools. The error bars show the mean and standard deviation from two experimental replicates. (Ordinary One-Way ANOVA; p=ns, ***, and **** indicate p>0.05, p< 0.001, and 0.0001 respectively). The error bars show mean and standard deviation. (C) Viability was determined by propidium iodide staining and the percent viability was measured relative to the viability of cells in DMSO control. (D) A heatmap of virus release per cell for the indicated clones (left: *vpr*^−^ pool 2 and right: *vpr*^+^ pool 2). Every column represents a unique cell clone’s response. The color bar indicates the extent of release in arbitrary units. Download FIG S3, TIF file, 1.3 MB. Copyright © 2022 Atindaana et al. 2022 Atindaana et al. https://creativecommons.org/licenses/by/4.0/ This content is distributed under the terms of the Creative Commons Attribution 4.0 International license.

10.1128/mbio.03748-21.4 FIG S4 (A and B) Sort gates for *vpr*-transduced *vpr*^−^ pools. Parental *vpr*^−^ pools were transduced with a Vpr expression vector. At 48 h posttransduction, cells were sorted into PE^+^ GFP^−^ and PE^+^ GFP^+^ cells. Flow plots show the gates for sorting of Vpr vector-transduced *vpr*^−^ pool 1 (left) and parental *vpr*^−^ pool 1 (right) (A) and Vpr vector-transduced *vpr*^−^ pool 2 (left) and parental *vpr*^−^ pool 2 (right) (B). Quadrants P6 and P5 were collected for further analysis. (C) Heat map of virus release per treated cell for *vpr^rev^*. The indicated clones in the first column are arranged from left to right by diminishing virus release per dual treatment. Pros, prostratin. Download FIG S4, TIF file, 1.7 MB. Copyright © 2022 Atindaana et al. 2022 Atindaana et al. https://creativecommons.org/licenses/by/4.0/ This content is distributed under the terms of the Creative Commons Attribution 4.0 International license.

Next, the behaviors of individual proviral clones within the populations were determined. Virus release from the treated cells was quantified by p24 equivalents, and cDNA was generated using virion genomic RNA upon LRA treatment. Zip codes were amplified from the viral cDNA and also from an untreated aliquot of the GFP^−^ cells’ DNA, and zip code libraries were high-throughput sequenced. The results were normalized to calculate the average virus release per treated cell for each clone (Fig. 5C; Fig. S3D).

This analysis revealed that many clones were not detectably reactivated. For example, of the 500 most abundant clones in the *vpr*^−^ pool, sequencing of virion RNA after reactivation treatment revealed no evidence of virus production for 102 of these abundant clones in the dually prostratin- and JQ1-induced virion cDNA pools. When cells were treated with both drugs, some clones in both the *vpr*^+^ and *vpr*^−^ pools displayed enhanced virus release per treated cell compared to single-treatment conditions. Interestingly, both proviral pools included a subset of clones that were not detectably reactivated by either prostratin or JQ1 when the LRAs were applied alone but that were reactivated upon dual-LRA treatment.

Surprisingly, and in only the *vpr*^−^ pool, the reactivation levels observed under dual-LRA conditions were lower than those observed with single-LRA use for a subset of clones (Fig. 5C, right, compare, for example, the top dozen rows in the *vpr*^−^ columns to those for the *vpr*^+^ clones). These same patterns of reactivation were observed when experiments were repeated using a second set of independently established *vpr*^−^ and *vpr*^+^ pools (Fig. S3D). A possibility suggested by this pattern of reactivation is that Vpr degrades a negative regulator of HIV-1 gene expression that is induced in Jurkat cells by prostratin treatment. Indeed, Vpr has been reported to cause the depletion or mislocalization of several factors that can repress HIV-1 expression, including class I histone deadenylases, the transcription factor ZBTB2, and the negative regulator of pTEFb, CTIP2 (64–67).

### Complementation of the *vpr*-defective pool led to depletion of high-LTR-active clones but did not discriminate against high-burst-size clones.

**FIG 6:**
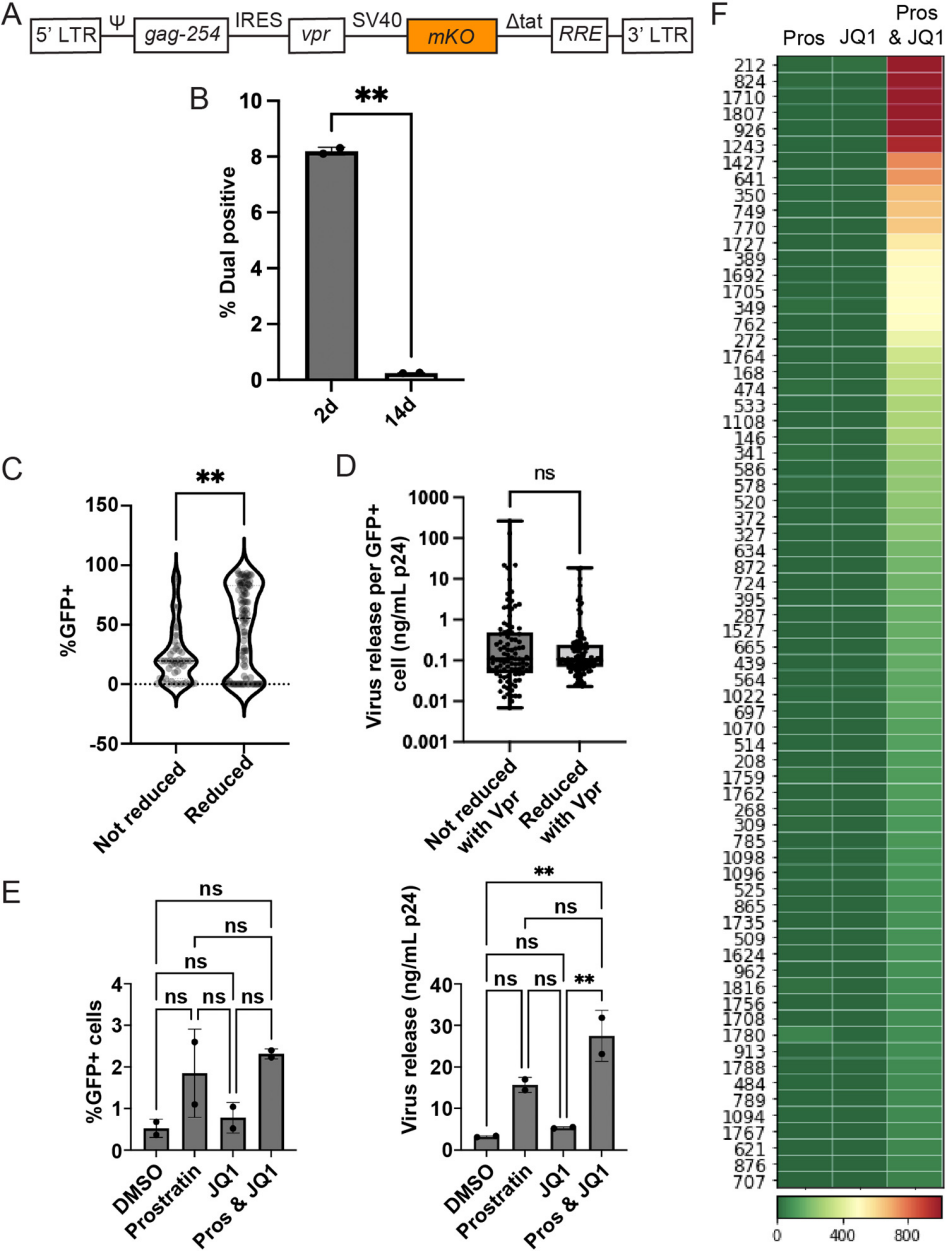
Complementation of *vpr*^−^ pools with a Vpr expression vector. (A) Schematic diagram of the Tat-deficient Vpr expression vector (not to scale). This vector was used to add *vpr* to *vpr*^−^ pools, and effects were determined by high-throughput analysis of the indicated viruses and cell samples. The vector contains *IRES-vpr* under the control of the HIV-1 LTR promoter and an SV40 promoter-driven mKO fluorescent reporter. (B) Population changes after *vpr* addition. Shown is a bar graph of the percentages of GFP^+^ cells for two polyclonal *vpr^rev^* pools established in parallel at day 2 (2d) and day 14 (14d) posttransduction (*P* = 0.0076 [significant pairwise comparison by a paired *t* test]). (C) Changes in clone sizes. Pairwise comparisons of %GFP^+^ values between the clones that were reduced (≥10-fold reduction in the relative abundance) and those that were not reduced (<1.5-fold reduction in the relative abundance) after *vpr* addition (significant pairwise comparison by a Mann-Whitney U two-tailed test). (D) Changes in clone sizes are not affected by viral burst sizes. Shown are pairwise comparisons of virus release per GFP^+^ cell between the clones that were reduced (≥10-fold reduction in the relative abundance) and those that were not reduced (<1.5-fold reduction in the relative abundance) after *vpr* addition (significant pairwise comparison by a Mann-Whitney U two-tailed test). (E) Bar graphs showing the percentages of GFP^+^ cells 24 h after reactivation with JQ1, prostratin, and dual treatment with prostratin-JQ1 for the *vpr^rev^* pool (left) and the *vpr*^−^ pool (right). (F) Heat map of the clonal (zip code) virus release per treated GFP^−^ cell of the *vpr^rev^* pool. Numbers at the left of each panel are clone identifiers generated by ordering proviral zip codes in decreasing relative abundances, as determined for the unsorted pools. Every row represents a unique cell clone’s response. The clones were ordered from top to bottom by diminishing virus release per treated cell upon dual-LRA treatment. The color bar indicates the extent of release per treated cell in arbitrary units. Cell density and other culture and assay conditions are the same as the ones described in the legend of Fig. 5.

As an additional test of the effects of *vpr* on polyclonal population composition, a functional copy of the *vpr* gene was added to cells harboring *vpr*^−^ proviruses at 16 days postinfection. This allowed a comparison of the behaviors of each of several hundred specific individual integrants in the presence or absence of *vpr*. This was achieved by transducing the *vpr*^−^ pool with a *tat*-deficient lentiviral vector containing LTR-driven *vpr*, which also contained the fluorescent marker *mKO* expressed from the constitutive simian virus 40 (SV40) promoter. This resulted in the expression of Vpr only when a preexisting *vpr*^−^ provirus was transcriptionally active. mKO (A monomeric version of the bioluminescent protein Kusabira Orange, which is derived from a Lithophyllon concinna gene) expression was used as a proxy for *vpr* vector transduction and enabled cells that were successfully transduced to be sorted in the phycoerythrin (PE) channel (Fig. 6A). At 48 h posttransduction, an unsorted aliquot was saved, while the remaining cells were sorted to identify GFP^−^ PE^+^ cells (Fig. S4A and B) for the work described below.

A comparison of the proportions of GFP^+^ cells in the parental *vpr*^−^ pool to the proportions in the same pool “reverted” to *vpr*^+^ by the introduction of the *vpr* vector (referred to here as the *vpr^rev^* pool) showed a marked decrease in GFP^+^ cells between day 2 and day 14 posttransduction (Fig. 6B). Although it cannot be ruled out that some feature of the transduction vector other than *vpr* influenced the cells’ properties, the selective loss of GFP^+^ transduced cells was consistent with predictions based on observations with the *vpr*^+^ pool, which showed that LTR-active cells were depleted in the presence of Vpr.

The proviral zip code abundances in unsorted *vpr*^−^ and *vpr^rev^* pools were then compared. Of the 500 most abundant clones in the *vpr*^−^ pool, zip codes were split into two groups based on the observed fold changes in relative abundance: those that were reduced by 10-fold or more in *vpr^rev^* (reduced clones) and those with no observed reduction in their relative abundance upon *vpr* addition (not reduced). A comparison between the “reduced” and “not-reduced” groups revealed that nearly all *vpr*^−^ pool clones that displayed high %GFP^+^ values, that is, clones in which most member cells displayed LTR activity, were reduced in the *vpr^rev^* pool (Fig. 6C).

In contrast, when the burst sizes of individual clones present in the *vpr^rev^* population, determined as described above for the parental *vpr*^−^ pool (Fig. 6D), were compared to those of the clones that were reduced versus those that were not reduced upon complementation, no differences in the amounts of virus released per active cell were detectable between groups.

The *vpr^rev^* pool was then used to address whether *vpr*^−^ pools rendered *vpr*^+^ by complementation displayed reactivation patterns similar to those of the original *vpr*^+^ pools. Transduced GFP^−^ *vpr^rev^* subpools were subjected to prostratin, JQ1, or dual treatment and analyzed by flow cytometry and virus release (Fig. 6E). In general, the magnitude of reactivation as measured by flow cytometry did not differ significantly compared to the no-drug control in the transduced cells (Fig. 6E, left). However, despite the significant increase in virus release for dually treated samples (Fig. 6E, right), the magnitude was diminished relative to that for the parental *vpr*^−^ GFP^−^ cells, with cells responding less to JQ1 treatment than they did before Vpr addition. Consistent with expectations if the phenotypes described above reflected Vpr, transduced cells showed enhanced reactivation upon dual-LRA treatment (Fig. 6E; Fig. S4C). Notably, clones (such as clones 53, 133, 161, 293, and 420, etc., in Fig. 6F) that were highly responsive to JQ1 treatment in the parental untransduced *vpr*^−^ pool and that differentiated the LRA responsiveness of the *vpr*^−^ pool from that of the *vpr*^+^ pool were highly represented among those lost upon *vpr* addition.

### Genomic features of persistent intact proviruses in patients on ART are more similar to those of *vpr*^+^ than to those of *vpr*^−^ pool members.

Although patients’ *vpr* genes are not routinely sequenced, it seems likely that most proviruses in HIV-1 patients on ART contain intact Vpr and have survived its selective pressures. Having observed differences between *vpr*^+^ and *vpr*^−^ viruses in cultured cells in terms of their reactivation patterns and proximity to genome features, we sought to determine which class of these proviruses more closely resembled those in persistent clinical isolates.

**FIG 7:**
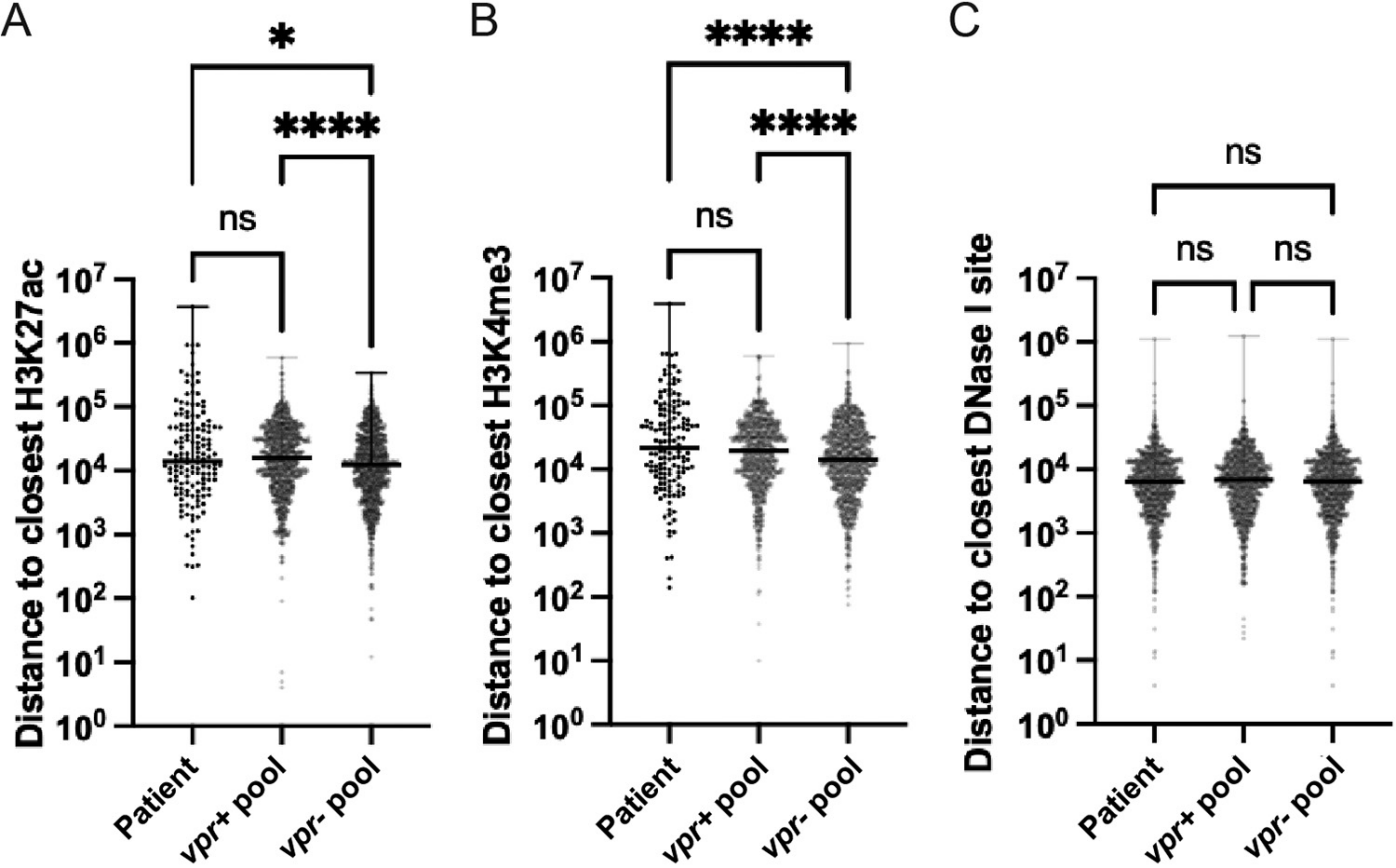
Comparison of patients’ integration site features with *vpr*^+^ and *vpr*^−^ pools. Previously published HIV integration sites from three patients were used to determine base distances to the closest H3K27ac, H3K4me3, and DNase I sensitivity sites. Distances were compared to those of the *vpr*^+^ and *vpr*^−^ pools. Box plots compare the base distances of *vpr*^+^, *vpr*^−^, and patients’ proviral integration sites to H3K27ac (means = 28,485, 22,228, and 89,771, respectively) (A), H3K4me3 (means = 32,989, 27,047, and 97,863, respectively) (B), and DNase I (means = 10,687, 10,236, and 10,191, respectively) (C) sensitivity sites (ns, *, **, ***, and **** indicate *P* values of >0.05, <0.05, <0.01, 0.001, and 0.0001, respectively, by a Kruskal-Wallis test).

To do this, previously published data on patients’ intact provirus integration sites (68) were analyzed for their proximity to H3K4me3, H3K27ac, and DNase I hypersensitivity sites that have been reported for Jurkat T cells and primary CD4^+^ T cells (see Materials and Methods) (63). When these proximities were compared to those in the *vpr*^−^ and *vpr*^+^ pools established here, prominent proviruses in the *vpr*^−^ pool, and not those in the *vpr*^+^ pool, were found to significantly differ from the patient proviruses in their proximities to H3K4me3 and H3K27ac marks (Fig. 7A and B; Fig. S5) but not DNase I hypersensitivity sites (Fig. 7C). These results suggested that the integration site distribution of persistent proviruses in patients across the tested genome marks was similar to those observed in *in vitro*-established *vpr*^+^ proviral populations but significantly different from what was observed within *vpr*^−^ proviral pools.

10.1128/mbio.03748-21.5 FIG S5 Comparison of patient integration site distances to the nearest H3K4me3 marks to those of the *vpr*^+^ and *vpr*^−^ pools. Previously reported integration sites of intact proviruses from three HIV patients were mapped to the nearest preexisting H3K4me3 mark in memory CD4^+^ T cells. Significant comparisons were determined by a Kruskal-Wallis test. ns, *, **, ***, and **** indicate *P* values of >0.05, <0.05, <0.01, 0.001, and 0.0001, respectively. Download FIG S5, TIF file, 0.3 MB. Copyright © 2022 Atindaana et al. 2022 Atindaana et al. https://creativecommons.org/licenses/by/4.0/ This content is distributed under the terms of the Creative Commons Attribution 4.0 International license.

## DISCUSSION

Here, we describe how Vpr disrupts polyclonal provirus population structures and alters the expression properties and latency-reversing agent responsiveness of residual proviral populations in cultured cells. The results illustrate the functional significance of HIV-1’s bimodal expression phenotypes in shaping proviral populations and show that whereas Vpr’s cytotoxicity will lead to the rapid depletion of clones constitutively or frequently expressing HIV-1 genes, *vpr*^+^ proviruses that are capable of supporting brief robust bursts of virion production can readily persist in proliferating infected cell populations *in vitro*.

Cell-based models are critical for HIV-1 persistence work, but they differ by cell type, the form of HIV-1 used, whether they use clones or polyclonal populations, and other parameters. Many experimental latency systems include reporter genes and/or delete viral genes believed to be unnecessary for silencing and reactivation (36, 37, 69). For example, two prominent studies that used barcoded proviruses to track virus dissemination in animals used *vpr*^−^ proviruses (36, 69). To address the consequences of such exclusions, the current study compared proviral populations with and without *vpr*. Note, however, that all vectors here also lacked *env* and *nef*, which are cytotoxic under some conditions and affect cell physiology in many ways, and thus, these omissions may have biased outcomes (70).

We have previously studied interclonal variation in HIV-1 gene expression using barcoded proviruses (53). That work revealed a broad range of expression variation among integrant clones. When virions produced by barcoded integrants were pseudotyped and polyclonal progeny were generated, the expression properties associated with specific barcodes in the first generation were lost, demonstrating that expression variation was nondeterministic and may be influenced by position effects. If similar variation exists *in vivo*, proviruses during suppressive ART may exist in epigenetic quasispecies upon which selection can act (71). Vpr was used as a source of selective pressure on infected cell populations here.

In our previous study, we compared the expression patterns of barcoded *vpr*^−^ proviruses in primary CD4^+^ T cells to those in Jurkat T cells and observed indistinguishable spectra of expression patterns (53). Although primary CD4^+^ T cells are a system closer to natural infection, these cells survive only 2 to 4 weeks *ex vivo* unless they are treated with antiapoptotic agents (48, 72) and do not proliferate unless they are stimulated in ways that can affect proviral expression patterns. Thus, because extended cell passaging was required and retaining expression patterns associated with initial integration events was desirable, we chose to work with Jurkat cells here, which limits the physiological relevance of our findings. Note, however, that Vpr reportedly exerts similar effects on primary CD4^+^ and Jurkat T cells (73).

Our and others’ works have shown that individual proviral clones can contain mixtures of LTR-active and -inactive cells (53, 74–76). Furthermore, the clonal progeny of individual infected cells can shift from being silenced to expressing their proviruses while maintaining overall proportions of cells with active proviruses (53, 74, 75). For example, in polyclonal *vpr*^−^ populations, individual member cells within each clone either express HIV-1 genes, as monitored by a GFP reporter, p24 staining, and virion release, or do not. Although some diminution of LTR expression is observed over time, for the most part, each clone adopts a stable, heritable pattern of bimodal gene expression (53).

Previous reports suggest that HIV-1’s bifurcating expression profiles exist in a minority of clones (54) and that stochastic fluctuations in gene expression enable probabilistic LTR-on/off fate decisions that are initially unstable but become stabilized by posttranscriptional feedback mechanisms (77). Our observations of bimodal HIV-1 expression patterns likely describe a similar phenomenon, although the majority of our clones display mixed expression phenotypes, and these phenotypes interchange over time in replicating cell populations, suggesting that they may exist in an oscillating circuit (53, 54, 78).

The zip-coded provirus approach used here enabled comparisons of *vpr*^+^ and *vpr*^−^ provirus-containing cell pools. Not surprisingly, *vpr*^+^ pools contained significantly fewer GFP^+^ cells (indicative of transcriptionally active proviruses) than did *vpr*^−^ pools. Dominant *vpr*^+^ clones included very few member cells that expressed HIV-1 genes (i.e., they were low-LTR-active clones), whereas high-LTR-active clones dominated the *vpr*^−^ pool.

These findings may help explain the inconsistencies in previous estimates of the fractions of HIV-1-infected cells that are transcriptionally active. Using polyclonal *vpr*^+^ proviruses *in vitro*, Dahabieh et al. suggested that most integrated proviruses are silent (79), even though it has been estimated that only a small fraction of infected patient cells are latent (32, 80). Consistent with the results of Dahabieh et al., we found that HIV-1 is transcriptionally inactive in most cells that persisted in *vpr*^+^ populations, with this reflecting the selective survival and relative amplification of low-LTR-active clones. However, the results showed that low-LTR-active clones with large burst sizes were not appreciably depleted. This finding is striking considering that GFP^+^ cells, cells that, in a binary sense, would score LTR active, displayed interclonal variation in burst size that spanned 4 orders of magnitude.

**FIG 8:**
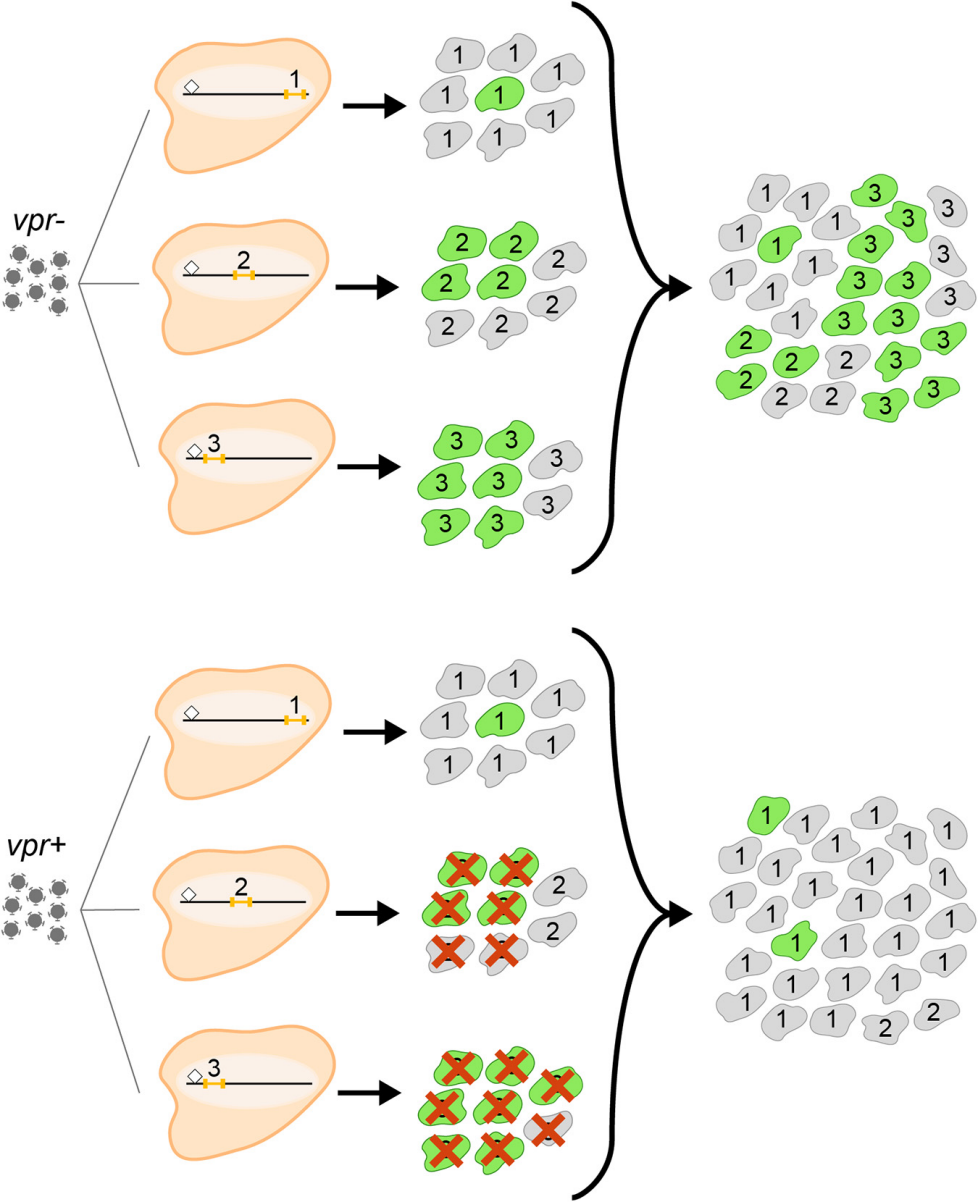
Schematic model of the effect of Vpr on the integrant populations’ proviral landscapes. From the left, proviruses integrate at indistinguishable genomic positions (designated 1, 2, and 3 above the small orange bars, which indicate proviruses) regardless of whether the infecting virus was Vpr^+^ or Vpr^−^. Note that integrants 1, 2, and 3 differ in their distances to the closest chromatin mark, which is indicated by a small diamond, which may affect their expression characteristics and responses to LRAs. Next, infected cells divide to form cell clones. For clone 1, cells that express HIV genes (indicated by green cells) are rare (low-LTR-active clone), and for other clones, more cellular members of each clone express HIV (e.g., clone 3 [high-LTR-active clone]). This pattern of bimodal gene expression is an intrinsic property of each clone (53). The red X’s indicate that high-LTR-active clone members are selectively depleted by Vpr. As a result, in the polyclonal populations shown at the right, low-LTR-active clones are enriched in the *vpr*^+^ pool.

These observations support the following model (Fig. 8). The initial integration site distribution will be Vpr agnostic, as previously demonstrated (55). As infected cells proliferate to form clones (13–15), some daughter cells will be LTR active, while other cells will be LTR inactive (53, 54, 78). Each clone will maintain a clone-specific equilibrium population of LTR-active versus -inactive cells over time, which is determined at least in part by integration site features. However, within clones, individual member cells’ phenotypes are transitory, with cells alternating between LTR-on and -off states (53, 54, 78). For high-LTR-active clones, most cells will be LTR active and subject to Vpr’s cytopathic effects. In contrast, for low-LTR-active clones, even clones that display very large burst sizes when cells are LTR active, clonal proliferation will proceed largely unimpeded.

The *vpr*^+^ and *vpr*^−^ proviral populations and their constituent clones here were compared for latency reactivation agent responsiveness, using LRAs selected based on their reported ability to induce HIV-1 to levels comparable to maximum reactivation by T cell activation (81). The overall pool trends for the *vpr*^+^ and *vpr*^−^ proviral populations were similar, although the responses differed in magnitude. In contrast, zip code analysis revealed that the behaviors of individual clones within the *vpr*^+^ and *vpr*^−^ populations differed. What caused the observed reduced levels of reactivation in the *vpr*^+^ pools was not determined. The widespread transcriptomic changes that Vpr induces in CD4 T cells may have played a role (33), and surviving LTR-inactive clones may have included defective proviruses. Another possibility is that the selective pressure exerted by Vpr may have depleted clones prone to high levels of reactivation. Consistent with this notion, proviruses farther from genome marks associated with active gene expression, a feature associated with *vpr*^+^ clones in the present study, are reportedly relatively resistant to reactivation (82). The adoption by the *vpr^rev^* pools of the *vpr*^+^ pool’s patterns of LRA responsiveness further corroborated the impact of Vpr on reactivation properties.

A comparison of integrants that dominated passaged *vpr*^+^ and *vpr*^−^ pools suggested that Vpr selects against proviruses proximal to genome marks associated with active gene expression. To address the relationship of these observations to persistent populations *in vivo*, we mapped the distances to preexisting marks for previously published integration sites of intact, inferred to be intact, or large proviruses in three patients on antiretroviral therapy (ART) (68). This analysis indicated that the proximities observed in patients were significantly more similar to those of *vpr*^+^ proviruses than to those of *vpr*^−^ pool members.

While proviral expression and latency have traditionally been viewed as mutually exclusive states, recent evidence, such as the selective depletion of patients’ intact proviruses over time and detectable HIV-1 RNAs in unstimulated latent cells, suggests that both basal expression and proviral inducibility may be heterogeneous, even within individual cell types (83, 84). Based on their study of replication-competent noninduced proviruses, Ho and colleagues noted that the expression of certain proviruses was infrequent despite T cell activation, suggesting that the induction of latent proviruses might be a stochastic process (19). Stochastic modeling coupled with laboratory experimentation in a separate study provided evidence that the transition from HIV-1 latency to viral outgrowth is a stochastic property (22). These examples of unanticipated expression patterns *ex vivo*, and their conclusions of HIV-1 expression stochasticity, were based in part on a failure to establish causal links between conditions believed to promote activation and its occurrence.

A possibly related but little-discussed problem observed with transcriptionally silenced *in vitro* latency models is that they tend to “spontaneously revert” during passage and generate small proportions of cells with transcriptionally active proviruses (75, 85). Our previous work and the findings here suggest an explanation slightly different from “spontaneous reversion.” Previous quantification of phenotypic switching within expression-sorted polyclonal populations demonstrated that the phenotypes of active and inactive subpopulations of proviral clones are not static (53). Instead, these phenotypes equilibrate such that most LTR-inactive cells are not stably silenced but rather represent the transient off-state of an on-off continuum. Thus, it seems conceivable that spontaneous reversion by a small subset of latency model cells, like the low proportion of GFP^+^ cells in the current study’s *vpr*^+^ pool, might more accurately be viewed as the programmed equilibration of a low-LTR-active clone than the outlier behavior of a subset of silenced cells.

Burst size variation of a magnitude similar to that described here is observed among patient samples reactivated *ex vivo* (23), and it seems likely that at least vestiges of bimodal expression are retained during natural infection. Weinberger and colleagues have proposed that HIV-1’s bifurcating expression phenotypes may represent a bet-hedging strategy, wherein some daughter cells support robust virus production while others remain quiescent, thus allowing clonal persistence whether or not the environment is conducive for viral expression (54). The selective pressures exerted by Vpr that were revealed in the present work may constitute one such determinant that specifies which proviruses will survive and contribute to persistent HIV-1 populations.

## MATERIALS AND METHODS

### Tissue culture and cell lines.

Two immortalized mammalian cell lines were used in this study. Human embryonic kidney HEK 293T and Jurkat cells were purchased from the ATCC (ATCC CRL3216 and ATCC TIB-152, respectively). Both cell lines were preserved as frozen stocks. HEK 293T cells were cultured in Dulbecco’s modified Eagle medium (DMEM) supplemented with 10% fetal bovine serum (FBS) (Gemini) and 125 μM gentamicin. Jurkat cells were cultured in RPMI 1640 medium supplemented with 10% FBS, 100 U/mL penicillin, 100 μg/mL streptomycin, 2 mM glutamine, and 55 μM β-mercaptoethanol. To propagate cells, frozen 1-mL aliquots of each cell line were thawed rapidly in a 37°C water bath and added to 9 mL of their respective prewarmed media. After mixing gently, cells were centrifuged at 2,500 rpm for 5 min. The supernatant was discarded, and the cell pellet was resuspended gently in prewarmed medium and plated in an appropriately sized tissue culture plate. HEK 293T cells were subcultured when confluence was between 75 and 100%. Jurkat cells were passaged 1/10 when the cell concentration reached 1 × 10^6^ cells/mL.

### Zip-coded vector and virus production.

Zip-coded vector DNA templates were generated by the digestion of previously described modified “inside-out” forms of GKO, which are called GPV^+^ and GPV^−^ here (53, 75), with ClaI and MluI. The resulting 11.4-kb DNA fragment, which was devoid of a plasmid backbone, was gel purified using a QIAquick gel extraction kit (catalogue no. 28706; Qiagen, Germantown, MD). A 304-bp insert fragment was generated by PCR with GPV^+^ or GPV^−^ as the template for *vpr*^+^ and *vpr*^−^, respectively; Phusion high-fidelity (HF) DNA polymerase (New England BioLabs, Inc., Ipswich, MA); and primers 5′-GACAAGATATCCTTGATCTGNNNNNNNNNNNNNNNNNNNNGCCATCGATGTGGATCTACCACACACAAGGC-3′ and 5′-CGGTGCCTGATTAATTAAACGCGTGCTCGAGACCTGGAAAAAC-3′. The 11.4-kb and 304-bp degenerate barcode-containing fragments were joined by Gibson assembly using HiFi DNA assembly mix (New England BioLabs) in a molar ratio of 1:5. Three micrograms of the resulting covalently closed circular DNA was directly cotransfected with 330 ng of pHEF-VSV-G (Addgene plasmid 22501) into a 70% confluent monolayer of HEK 293T cells in a 10-cm dish using polyethylenimine (Polysciences, Inc., Warrington, PA) at 4× total transfected DNA in 800 μL 150 mM NaCl. DMEM was replaced with 4 mL RPMI 1640 medium with 10% FBS and 1% penicillin-streptomycin (Pen/Strep) at 24 h posttransfection, and culture medium was harvested by filtering through a 0.22-μm filter (catalogue no. 09-720-511; Fisher Scientific) at 48 h posttransfection.

### Generation of zip-coded proviral pools.

A total of 1,000 μL of virus-containing medium was mixed with Polybrene at a final concentration of 0.5 μg/mL and brought to a total volume of 2,000 μL by the addition of RPMI 1640. This infection mixture was added to 5 × 10^6^ Jurkat cells and incubated in two wells of a 12-well plate at 37°C with 5% CO_2_ for 5 h. Infected cells were then transferred to 10-mL Falcon tubes and centrifuged for 5 min at 2,500 rpm at 4°C. Following centrifugation, the supernatants were replaced with fresh medium, and cell pellets were resuspended and cultured in two wells of a 6-well plate at 37°C with 5% CO_2_. At 24 h postinfection, puromycin was added to a final concentration of 0.5 μg/mL. The infected cells were expanded into 6-cm culture plates without puromycin on day 5. At 10 days postinfection, the culture supernatant was replaced with fresh medium, and the cultures were divided into aliquots to be either frozen, prepared for integration site sequencing, or further expanded for four additional days and sorted into GFP^+^ and GFP^−^ subpools for subsequent experiments. Puromycin-resistant colony-forming titers of pseudotyped barcoded virus stocks were determined by infection of HEK 293T cells, and equivalent infectious units were used to generate *vpr*^+^ and *vpr*^−^ Jurkat cell pools.

### Isolation of clonal populations.

Single cells were isolated from the above-described infected Jurkat cell libraries by limiting dilution and expanded to generate clonal populations. The purity of clones was verified by zip code. Briefly, a 555-bp U3-R PCR product was amplified from cellular DNA extracted from each clonal population using the primers 5′-ACGAAGACAAGATATCCTTGATC-3′ and 5′-GCACTCAAGGCAAGCTT-3′, which flank the zip-coded region. PCRs used Q5 Hot Start high-fidelity 2× master mix (New England BioLabs) according to the manufacturer’s protocol, for 30 cycles with a 1-min extension step at 72°C and a 60°C annealing temperature. PCR amplicons were gel purified using a QIAquick gel extraction kit (catalogue no. 28706; Qiagen, Germantown, MD) and submitted to Genewiz (South Plainfield, NJ) for Sanger sequencing with 5′-GCACTCAAGGCAAGCTT-3′. The results revealed that clones 1, 2, and 3 (Fig. 1B) contained the zip codes AATACAAGTCGGACCACCTG, GTAACCTTGGCGTCAGGAG, and GTGATGGTAGCGACAGCGTG, respectively.

### Construction of the *vpr*^+^ lentiviral vector and its use.

A 2,933-bp *in vitro* DNA-synthesized *IRES-vpr mKO* fragment was ordered from Genewiz (South Plainfield, NJ) using an internal ribosome entry site (IRES) sequence from pTRIPZ-hDDX5/7 (Addgene plasmid 71307) (86) and *vpr* and *mKO* from GPV^+^. An HIV-1 lentiviral vector fragment was generated by XbaI and MfeI digestion of pWA18puro (87) to remove its puromycin resistance cassette and was gel purified using a QIAquick gel extraction kit (catalogue no. 28706; Qiagen, Germantown, MD). The resulting DNA fragments were joined by Gibson assembly using HiFi DNA assembly mix (New England BioLabs) according to the manufacturer’s protocol to generate plasmid pEA216-1. Ten micrograms of pEA216-1 was then cotransfected with 5 μg of the pCMVΔR8.2 helper plasmid (Addgene plasmid 122263) and 1 μg of pHEF-VSV-G into 70% confluent monolayers of HEK 293T cells in a 10-cm dish using polyethylenimine (Polysciences, Inc., Warrington, PA) at 4× total transfected DNA in 800 μL 150 mM NaCl. DMEM was replaced with 4 mL RPMI 1640 medium with 10% FBS and 1% Pen/Strep at 24 h posttransfection, and culture medium was harvested by filtering through a 0.22-μm filter (catalogue no. 09-720-511; Fisher Scientific) at 48 h posttransfection. Parental *vpr^−^* pools were infected with this filtered medium and sorted for mKO and GFP expression at 48 h posttransduction to generate *vpr^rev^* pools. Vpr expression was evaluated by cell death. All mKO^+^ cells were assumed to contain the Tat-inducible *vpr* vector. Therefore, the survival of mKO^+^ cells (PE) compared to dually positive cells (see Fig. S4A and B, left panels, in the supplemental material) suggested that in the absence of Tat from a preexisting provirus, uninduced Vpr expression was not pronounced enough to kill at least most of the transduced cells. However, the possibility that low levels of uninduced Vpr had led to the depletion of some singly positive cells cannot be ruled out.

In determining the effect of Vpr addition on parental *vpr^−^* pools, zip code abundances in sequences amplified from genomic DNA of parental *vpr*^−^ unsorted cells and from Vpr expression vector-transduced *vpr^rev^* mKO^+^ cells were compared. Two categories of zip codes were defined: reduced zip codes were those that were reduced 10-fold or more after Vpr transduction, whereas those with a change of 1.5-fold or less in their relative abundances were regarded as not being affected by *vpr* addition (not reduced).

### Flow cytometry and cell sorting.

For fluorescence-activated cell sorter (FACS) analysis by flow cytometry, Jurkat cells were suspended in phosphate-buffered saline (PBS) containing 1% FBS (FACS buffer). Dead cells were excluded from all analyses and sorting experiments using propidium iodide (PI). Acquisition was carried out on the fluorescein isothiocyanate (FITC) channel for GFP and on the PE channel for PI. Cell fluorescence was assessed using a BD LSR Fortessa instrument (BD Biosciences), and data were analyzed using FlowJo software, version 10.6 (FlowJo, LLC, Ashland, OR). Infected cells were sorted into GFP^+^ and GFP^−^ subpopulations by flow cytometry using a FACSAria II (BD Biosciences, Franklin Lakes, NJ) or an iCyt Synergy SY3200 (Sony Biotechnology, San Jose, CA) cell sorter at the flow cytometry core of the University of Michigan.

### Latency-reversing agents and reactivation.

JQ1 and prostratin were purchased from Sigma-Aldrich. Each LRA was dissolved in DMSO (Thermo Fisher) to produce stocks. For each experiment, stocks were added to culture medium to achieve final concentrations of 2 μM JQ1 and 10 μM prostratin. Dual-LRA treatment was performed by adding the two LRAs to the same culture medium to achieve their respective single-LRA concentrations. For reactivation experiments, GFP^−^ cells sorted on day 14 postinfection were cocultured with the appropriate LRA for 24 h. Cells were then centrifuged at 2,000 rpm at 4°C for 5 min, and cells pellets were washed twice with ice-cold FACS buffer after being stained with PI for 5 min at room temperature. The resulting cells were then washed and assessed by flow cytometry, and the p24 concentration in the culture supernatants was determined by a reverse transcriptase (RT) assay.

### Zip code sequencing libraries.

Zip codes were amplified from the genomic DNA of infected cells as well as from the RNA of virus released into cell media. The generation of zip-coded sequencing libraries from infected cells was initiated by harvesting DNA from an aliquot of 2 × 10^6^ infected cells. Genomic DNA extraction was carried out using the Qiagen (Germantown, MD) DNeasy blood and tissue kit. Zip codes were then amplified by PCR from 200 ng of the DNA template using Phusion high-fidelity DNA polymerase (New England BioLabs) in HF buffer. Primers were designed to flank the zip code region (primer sequences 5′-NNACGAAGACAAGATATCCTTGATC-3′ and 5′-NNTGTGTGGTAGATCCACATCG-3′). Multiple copies of these primers were created, each with a unique pair of known, randomized nucleotides at the 5′ end, to confirm that no cross-contamination had occurred between samples. Reaction mixtures were cycled 29 times with a 30-s extension step at 72°C and a 59°C annealing temperature. Zip code amplicons were purified using the DNA Clean and Concentrator-5 kit (catalogue no. D4013; Zymo Research, CA) and eluted in 15 μL of MilliQ H_2_O. To amplify zip codes from virus, a tissue culture plate of infected cells was decanted into a conical tube and centrifuged at 2,500 rpm for 5 min. The virus-containing medium was separated from the cell pellet and passed through a 0.22-μm filter. To concentrate the virus, medium was subjected to ultracentrifugation (25,000 rpm) for 120 min through a 20% sucrose cushion. Viral pellets were then resuspended in 200 μL PBS, and viral RNA was extracted using the Quick-RNA viral kit (catalogue no. R1034 and R1035; Zymo Research, CA) according to the manufacturer’s protocol and eluted in 10 μL RNase-free water. cDNA was synthesized using 5 μL of the eluent as the template, using the U3 antisense primer 5′-TGTGTGGTAGATCCACATCG-3′ and Moloney murine leukemia virus (M-MLV) RT RNase (H^−^) (catalogue no. MR3681; Promega, WI) according to the manufacturer’s protocol. Zip codes were amplified from this cDNA under the conditions described above. The zip code amplicons were then used to generate MiSeq libraries for sequencing as described previously (53).

### Integration site determination.

Genomic DNA was extracted from *vpr*^+^ and *vpr^−^* cells at 10 days postinfection using the Qiagen DNeasy blood and tissue kit (Qiagen), and 200 ng of DNA was sheared to 1-kb fragments using an M220 instrument and microTUBE according to the manufacturer’s recommended settings (Covaris, Woburn, MA). HIV-1 insertion site libraries were prepared and sequenced using methods described previously (53).

### Quantification of virus release.

Virion production was quantified using a real-time reverse transcription-PCR assay developed previously by Pizzato et al., as modified by Kharytonchyk et al. (87, 88). Briefly, viral lysates were prepared by adding 5 μL of the culture supernatant to 5 μL of lysis buffer. Using MS2 RNA as the template, MS2 cDNA was synthesized with viral lysates and quantified by real-time PCR in one reaction. Released virus was quantified and normalized for p24 levels based on values determined in parallel for reference samples.

### Zip code quantification and analysis.

Zip codes were identified and quantified from Illumina sequencing reads using a previously described custom suite of tools implemented in Python (https://github.com/KiddLab/hiv-zipcode-tools). Briefly, 2- by 75-bp paired reads were merged using flash v1.2.11 (73). Zip codes were identified by searching for known flanking sequences (with up to 1 mismatch). Only candidate zip codes with a length of 17 to 23 nucleotides were considered, and the read count for each unique zip code was tabulated. To identify sets of zip codes for further analysis, zip code families, which account for PCR and sequencing errors, were determined by clustering together the observed unique zip codes. Abundances for the zip codes were then determined by assigning unique zip codes to the most abundant family whose sequence was within 2 mismatches and summing their associated read counts. Only zip code families with corresponding data in the integration site data were selected for further analysis.

For each latency reversal treatment condition, clonal virus release was determined by multiplying the fractional abundances of zip codes from the cDNA sequencing libraries of each treatment by their corresponding sample’s pool p24 concentration, as measured 24 h after LRA treatment. The resulting clonal p24 values for each zip code in prostratin-, JQ1-, and combination prostratin-JQ1-treated samples were divided by clonal p24 values defined for cell samples exposed to only DMSO to determine fold changes after LRA treatment.

The burst sizes of clones were determined by multiplying the fractional abundances of zip codes from the cDNA library of the unstimulated sample’s pool p24 concentration (basal viral release). The resulting p24 values for each zip code were then divided by their corresponding fraction of abundances determined in the gDNA sequencing library of GFP^+^ sorted cells from the same unstimulated sample from which the cDNA library was made.

The %GFP^+^ value for each zip code was determined as *F_i_* = [(*G_i_* × *P*)/(*G_i_* × *P* + *W_i_* × *Q*)] × 100 (53), where *F_i_* is the GFP^+^ percentage of zip code *i*, *G_i_* is the fraction abundance of zip code *i* in the GFP^+^ sorted pool, *W_i_* is the fraction abundance of zip code *i* in the GFP^−^ sorted pool, *P* is the fraction of cells that were sorted into the GFP^+^ pool, and *Q* is the fraction of cells that were sorted into the GFP^−^ pool.

### Determination of chromatin marks.

H3K27ac marks annotated for the Jurkat E-6-1 clone were sourced from ChIP-Atlas (http://chip-atlas.org/view?id=SRX1041803), H3K4me3 and DNase I sensitivity site data sets were downloaded from the ENCODE Project (https://www.encodeproject.org/) with Sequence Read Archive accession no. SRX1041803 and ENCODE Project identifiers ENCFF304GVP (https://www.encodeproject.org/experiments/ENCSR000EOS/) for genome marks preexisting in Jurkat cells, iENCFF341XUX (https://www.encodeproject.org/experiments/ENCSR807WEO/), and ENCFF053LHH (https://www.encodeproject.org/experiments/ENCSR724GUS/) (63). Bedtools was then used to map the distance to the closest known annotated marks. Analysis of the proximity to these genome marks was done using the matplotlib and scipy.stats packages in Python, and the results were exported into GraphPad Prism version 9.1.2 to plot graphs.

### Data availability.

All sequence data have been deposited to the SRA under accession no. SRX1041803.

10.1128/mbio.03748-21.6 TABLE S1 Table of zip codes, these clones’ fractions of abundance, and integration sites of *vpr*^−^ and *vpr*^+^ pools. Download Table S1, XLSX file, 0.2 MB. Copyright © 2022 Atindaana et al. 2022 Atindaana et al. https://creativecommons.org/licenses/by/4.0/ This content is distributed under the terms of the Creative Commons Attribution 4.0 International license.
