# Supplementary figures and images for: Bimodal Expression Patterns, and Not Viral Burst Sizes, Predict the Effects of Vpr on HIV-1 Proviral Populations in Jurkat Cells
Source: mBio. 2022 Apr 6;13(2):e03748-21. doi: 10.1128/mbio.03748-21 (PMC9040753; doi:10.1128/mbio.03748-21)

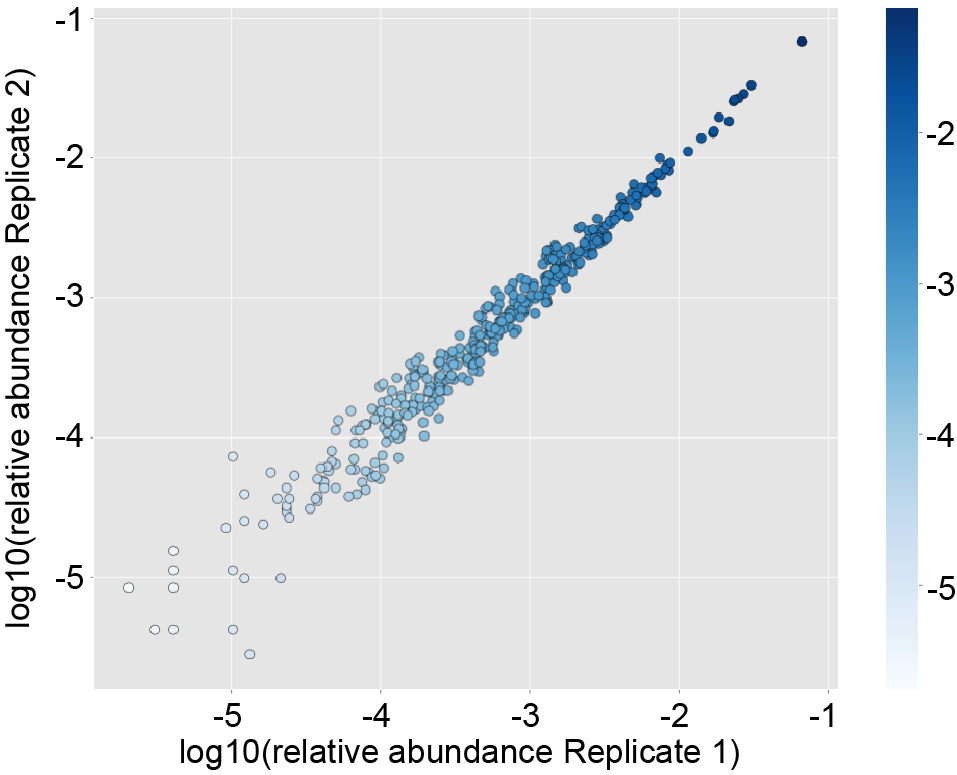

Supplement: FIG S1 [file mbio.03748-21-sf001.tif]

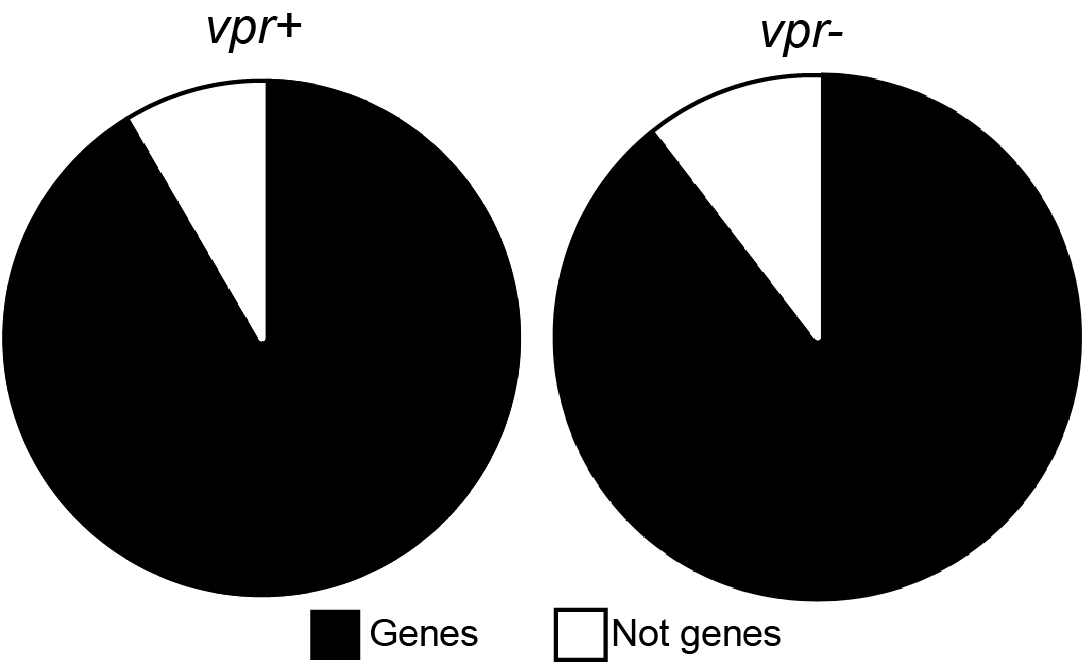

Supplement: FIG S2 [file mbio.03748-21-sf002.tif]

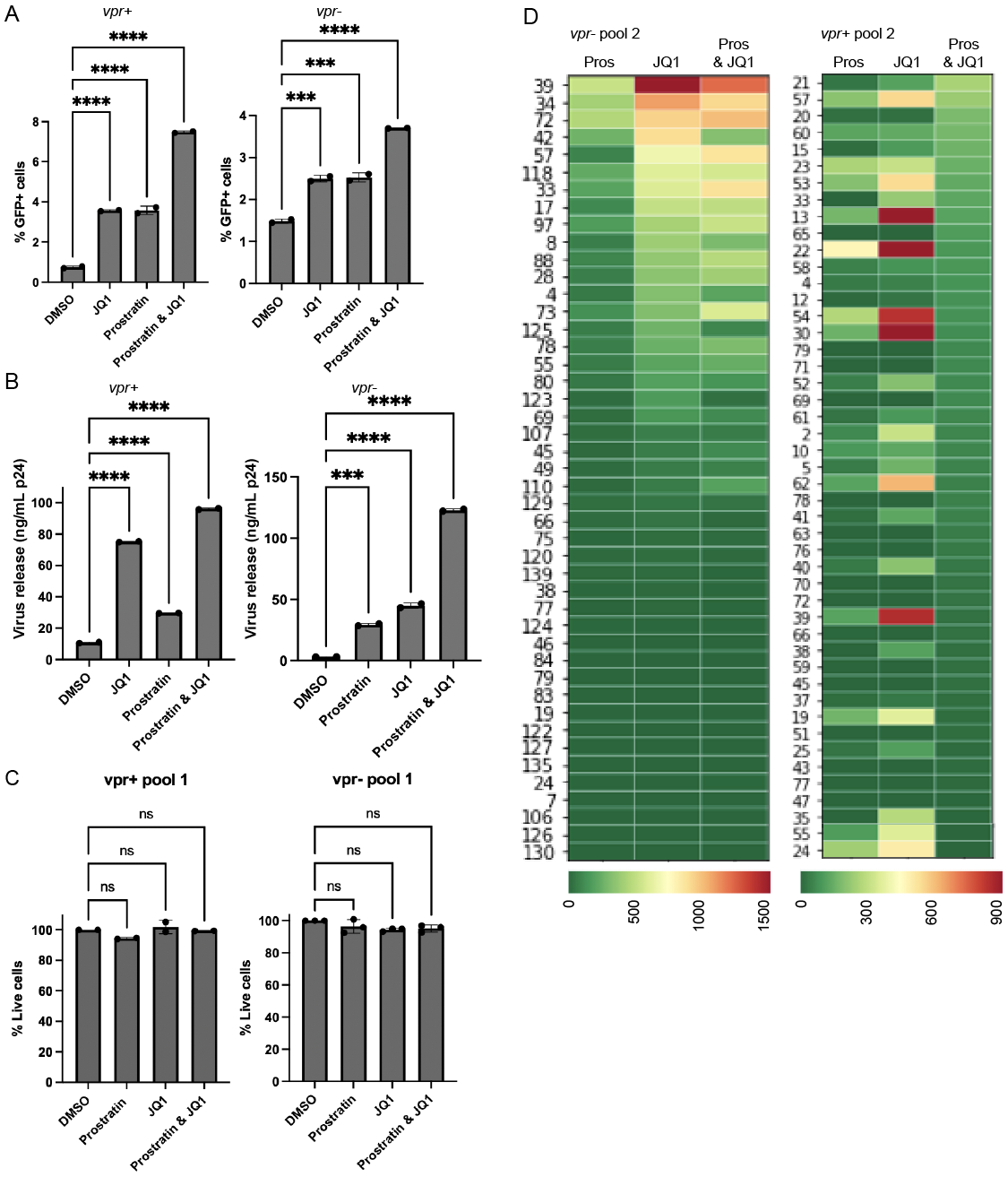

Supplement: FIG S3 [file mbio.03748-21-sf003.tif]

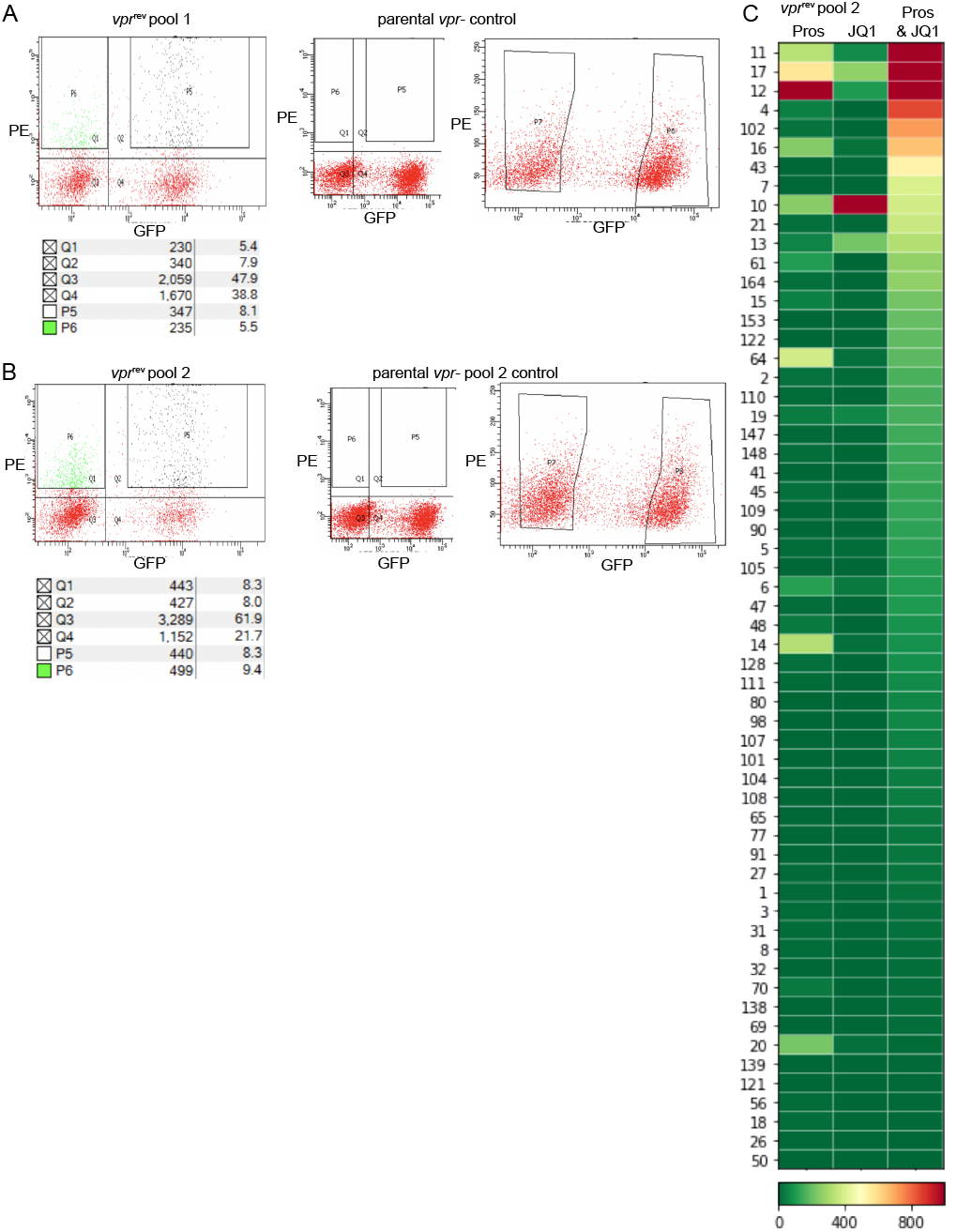

Supplement: FIG S4 [file mbio.03748-21-sf004.tif]

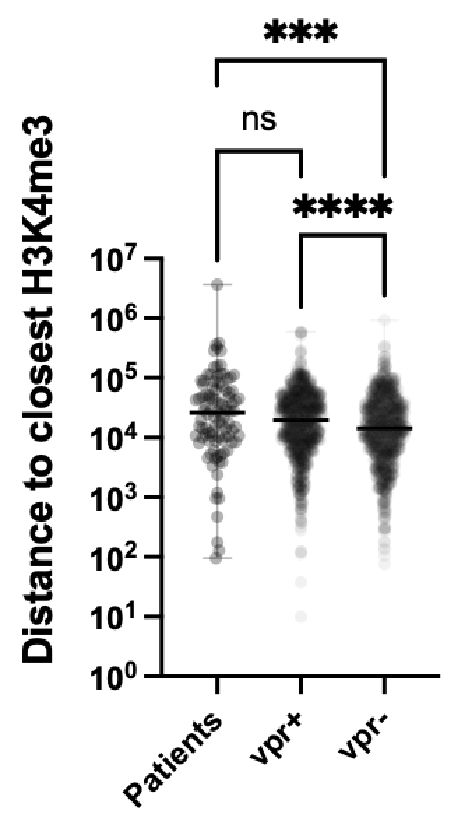

Supplement: FIG S5 [file mbio.03748-21-sf005.tif]
